# Sulfur radical formation from the tropospheric irradiation of aqueous sulfate aerosols

**DOI:** 10.1073/pnas.2202857119

**Published:** 2022-08-29

**Authors:** James D. Cope, Kelvin H. Bates, Lillian N. Tran, Karizza A. Abellar, Tran B. Nguyen

**Affiliations:** ^a^Department of Environmental Toxicology, University of California, Davis, CA 95616;; ^b^Center for the Environment, Harvard University, Cambridge, MA 02138;; ^c^Department of Chemistry, University of California, Davis, CA 95616

**Keywords:** aerosol, sulfur, radical, sulfate

## Abstract

The sulfate anion radical (SO_4_^•–^) is known to be formed in the autoxidation chain of sulfur dioxide and from minor reactions when sulfate or bisulfate ions are activated by OH radicals, NO_3_ radicals, or iron. Here, we report a source of SO_4_^•–^, from the irradiation of the liquid water of sulfate-containing organic aerosol particles under natural sunlight and laboratory UV radiation. Irradiation of aqueous sulfate mixed with a variety of atmospherically relevant organic compounds degrades the organics well within the typical lifetime of aerosols in the atmosphere. Products of the SO_4_^•–^ + organic reaction include surface-active organosulfates and small organic acids, alongside other products. Scavenging and deoxygenated experiments indicate that SO_4_^•–^ radicals, instead of OH, drive the reaction. Ion substitution experiments confirm that sulfate ions are necessary for organic reactivity, while the cation identity is of low importance. The reaction proceeds at pH 1–6, implicating both bisulfate and sulfate in the formation of photoinduced SO_4_^•–^. Certain aromatic species may further accelerate the reaction through synergy. This reaction may impact our understanding of atmospheric sulfur reactions, aerosol properties, and organic aerosol lifetimes when inserted into aqueous chemistry model mechanisms.

## Significance

It was found that shining natural or artificial sunlight on concentrated solutions of sulfate ions mixed with organics, a mixture commonly found in atmospheric aerosol particles, can generate sulfur-containing radicals under a variety of conditions. This reaction has not previously been characterized in atmospheric chemistry. These reactive radicals can subsequently degrade organic compounds in atmospheric particles, forming a variety of products that stay in the particle water and small molecules that are volatile enough to partition to the gas phase. In particular, this source of sulfur radicals can produce surface-active organosulfates and organic acids.

Atmospheric aerosol particles, which are mixtures of organics, inorganics, water, and other components suspended in air (1), are critical drivers of air pollution and short-term forcers of climate impacts (2). Sulfate anions (SO_4_^2–^, HSO_4_^−^) are ubiquitous in aerosols. With mass fractions of >40% in several areas, sulfate is the largest anthropogenic contributor to fine-mode aerosols (1). Under typical atmospheric conditions, ionic strengths of sulfate in aerosol water are high [e.g., >4 M predicted at less than 80% relative humidity (RH) (3)]. We found that these high aqueous sulfate activities can induce considerable chemistry through the formation of sulfate anion radicals (SO_4_^•–^), and potentially other sulfur oxy radicals, under solar light available in the troposphere in a chemical reaction that has yet to be accounted for in atmospheric model mechanisms.

Much of the discussion of SO_4_^•–^ formation in atmospheric clouds, fogs, and aerosol water is centered on the autoxidation chain of sulfur dioxide (SO_2_) that is emitted from fossil fuel combustion (4). SO_4_^•–^ is formed when S(IV) species are oxidized, e.g., by the hydroxyl (OH) radical. There are also small sources of SO_4_^•–^ radicals from the reactions of bisulfate (HSO_4_^−^) with OH, or HSO_4_^−^/SO_4_^2–^ with NO_3_ (5). Once formed, SO_4_^•–^ may react with organics through electron transfer, addition to double bonds, and H-abstraction (6). The ability for SO_4_^•–^ to form covalent C-O-S bonds through addition (7) strongly contribute to organosulfate formation in the atmosphere (8). While SO_4_^•–^ reactions with organics are included in computational models of atmospheric aerosol chemistry, their importance has been considered limited due to the minor SO_4_^•–^ source strength of known reactions and the notion that other highly reactive radicals (OH, NO_3_) are needed to initiate SO_4_^•–^ chemistry (4, 9).

Yet, prior studies provide evidence of unexplained sulfur chemistry that did not result from OH or NO_3_ radicals when sufficient ionic strength of sulfate ions is used. Nozière and coworkers (10) observed the formation of organosulfate compounds when high-ionic-strength solutions of ammonium sulfate (AS) were irradiated with UV B (UVB) radiation in the presence of alkenes and in absence of radical sources. However, without a clear mechanism of formation, they attributed the phenomenon to the HSO_4_^−^ + OH reaction. It is not clear if the pH of the solutions under study would allow a sufficient population of HSO_4_^−^ to exist, nor from where the OH radicals originate. The authors speculate that the organics in the solution produce OH radicals via unknown pathways. At approximately the same time, Galloway and coworkers (11) showed that glyoxal uptake onto wet AS particles produce organosulfates, but only when the particles were irradiated with UVA light, not in the dark. While glyoxal is known to react with sulfate anions in the dark, it is not clear why light was needed to produce organosulfates. We now understand these prior observations in the context of new data: SO_4_^•–^ radicals are produced when AS solutions and AS particle water are irradiated together with organics using light available in the solar spectrum. However, these reactions cannot be attributed to any known formation pathways of SO_4_^•–^. Rather a new reaction needs to be invoked; the exact mechanism at this point remains elusive.

## Results

### Reactivity of Bulk-Phase Aqueous AS with Various Chemical Systems When Irradiated with Artificial and Natural Light.

Solutions of AS at various ionic strength that were mixed with organic reagents and irradiated by UVB light (*SI Appendix*, Fig. S1) degraded all of the organic compounds tested significantly faster than direct photolysis by the same light source. Chemical systems that were tested were chosen to represent environmentally relevant organics (*Materials and Methods*, and references therein), including: isoprene’s 2,3-dihydroxy 1,4-dinitrates (DHDN), *p*-nitrophenol (p-NP), pinonic acid (PA), 2,4-dinitrophenylhydrazine (DNPH), the formic acid adduct of DNPH (FADNPH), 1,2-dihydroxyisoprene (1,2-DHI), erythritol, and 1,3,5-trihydroxybenzene (THB). These compounds are relevant to biogenic, biomass burning, and urban aerosols. Furthermore, they contain a diversity of functional groups (e.g., -ONO_2_, -OH, -COOH, -C = C-, phenyl). For the majority of experiments, a 3.7 M ionic strength solution of AS ((NH_4_)_2_SO_4_) was studied to compare to Nozière et al. (10), at an unadjusted pH of 5–6.

**Fig. 1.**
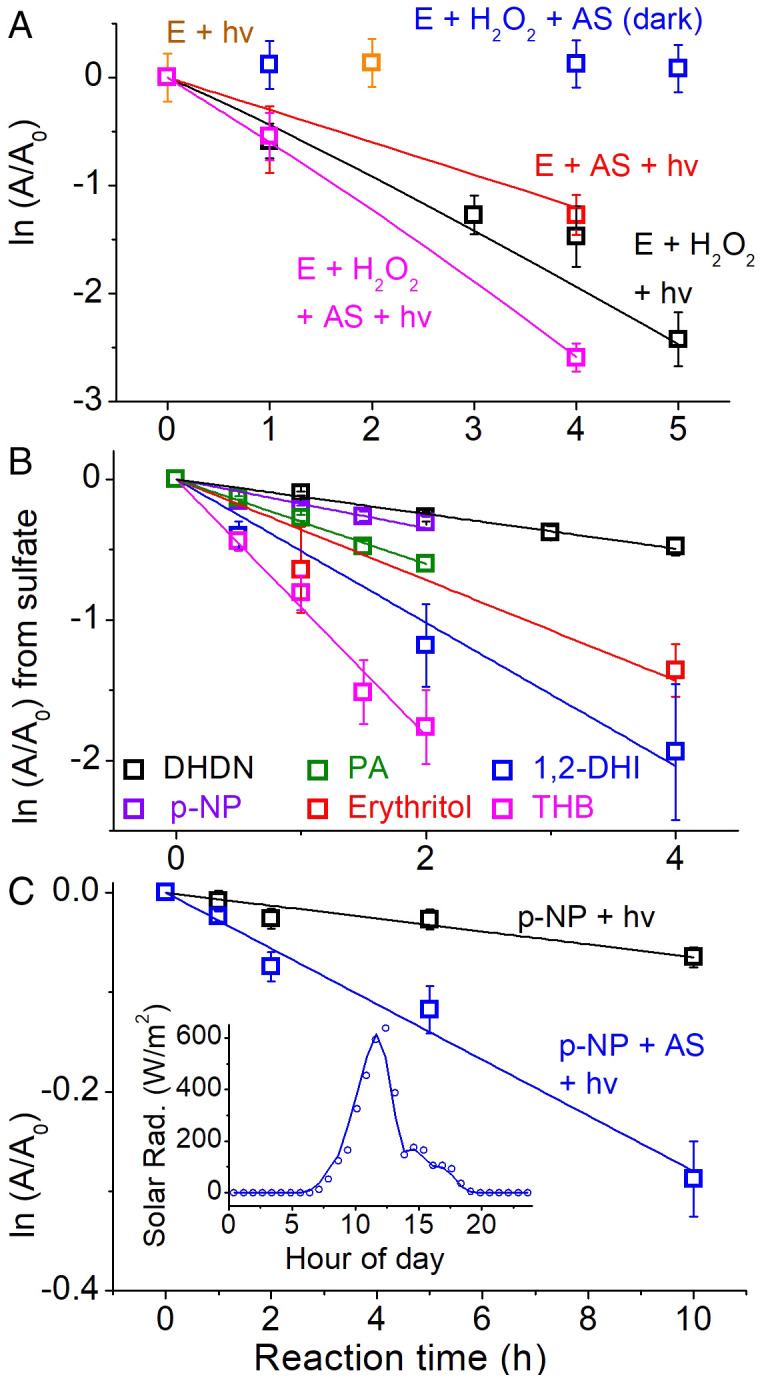
(*A*) Degradation of erythritol (E) from photochemical and dark reactions in the laboratory, plotted as the natural log of the remaining fraction of analyte (A/A_0_); (*B*) degradation of environmentally relevant organics from the photochemistry of 3.7 M reagent-grade AS in the laboratory, after subtraction of direct photolysis; (*C*) degradation of p-NP in sunlight on a wildfire-impacted day as monitored by HPLC-HRMS. Organic concentrations are 1 mM. Solid lines are model simulations in *A*, and least-squares fits in *B* and *C*. Solar radiation data (insert in *C*) are obtained from the KCASACRA441 weather station (38.63°N, 121.36°W) nearby in Sacramento, CA.

Fig. 1*A* shows that a UV-irradiated solution of 3.7 M reagent grade (RG) AS mixed with erythritol will degrade erythritol almost as quickly as reactions using aqueous OH (H_2_O_2_ + light). Steady-state concentrations of OH used (∼7 × 10^−14^ M) were chosen to be higher than those measured in aerosol or cloud water [< 1 × 10^−14^ M (12–14)] but comparable to some modeled values (15, 16). The OH experiment degraded erythritol with a first-order time constant of ∼0.45 h^−1^ (Fig. 1*A*, black) and lifetime (τ) of ∼2 h. The AS + hv experiment produced a time constant of 0.35 h^−1^ (τ ∼ 3 h), consistent with ∼2 × 10^−12^ M of steady-state SO_4_^•–^ radicals assuming known rate coefficients (4). The experiment with organic + AS + H_2_O_2_ + light degraded organics faster than either AS + light or H_2_O_2_ + light alone, suggesting that OH and SO_4_^•–^ reactivities do not cancel. Direct photolysis and dark reactions are negligible (Fig. 1*A*, orange and blue).

The slowest reaction of the organics we examined in irradiated AS solution is attributable to the aliphatic DHDN and the fastest to the aromatic THB (Fig. 1*B*). Kinetic coefficients for all experiments are reported in *SI Appendix*, Table S1. These observations are consistent with the general trends of SO_4_^•–^ reactions, which are more selective than OH. For aliphatic systems, the rate coefficients with SO_4_^•–^ can be >10 times slower than that of OH, but for some aromatic systems they are comparable (4). DHDN also reacts slowly with OH (17); thus, the data are consistent with the current understanding. The photoinduced AS chemistry with organics is also active under natural sunlight at sea level (Fig. 1*C*), even during a hazy wildfire-smoke-impacted day.

Control experiments were performed to rule out the possibility that chromophoric organic impurities and transition metal impurities are responsible for the chemistry. For example, chromophoric organics can produce triplet intermediates that are also oxidants (18) and a photoactive iron-sulfate complex can produce SO_4_^•–^ through irradiation, but with low quantum yields of SO_4_^•–^ and with accompanying OH formation from iron-hydroxide complexes (19, 20). Two grades of AS, reagent grade (RG) and molecular biology (MB) grade, were used for experiments, for which RG has a small amount of absorbing impurities, but MB did not (*SI Appendix*, Fig. S2). These two grades of AS have been quantified to have low levels of trace metal impurities (*SI Appendix*, Table S2), but RG has more trace iron and aluminum. However, both grades of AS resulted in similar kinetic decays for nitroaromatics and PA under laboratory (*SI Appendix*, Fig. S3) and sunlight (*SI Appendix*, Fig. S4) irradiation, suggesting that variations in impurity levels are not consequential for the reactions of the tested organics. Control experiments with metal chelate ethylenediaminetetraacetic acid (EDTA) in a large excess resulted in an identical first-order loss rate compared to experiments without EDTA (*SI Appendix*, Fig. S5), further suggesting a negligible contribution of transition metal impurities to the AS photochemistry.

Using a kinetic model (*Materials and Methods* and *SI Appendix*, Table S3), we can adequately fit the results of the 200 mM H_2_O_2_ + organic + hv experiment (Fig. 1*A*, black line) from experimental inputs and known aqueous H_2_O_2_ photolytic parameters (21). However, the results for the 3.7 M AS + organic + hv chemistry (Fig. 1*A*, red line) cannot be fit with all of the S(VI) reactions of CAPRAM (5), a state-of-the-art aqueous model, or any other known reaction in the literature. A photochemical SO_4_^•–^ source relating to the sulfate anion itself is needed. Yet, the precise way to represent this reaction in models is unclear at this point. Radical production appears to be significant; however, radical quantum yields are still unknown and SO_4_^•–^ + organic kinetic parameters are missing for many of the environmental compounds studied here, which preclude a full environmental assessment. It is also possible that SO_4_^•–^ reaction with aromatics induced an autoxidation (22), which can accelerate decay of aromatics like THB. These synergistic phenomena require additional research.

### Evidence for Sulfur Radical Production from Irradiated Sulfate Solutions.

**Fig. 2.**
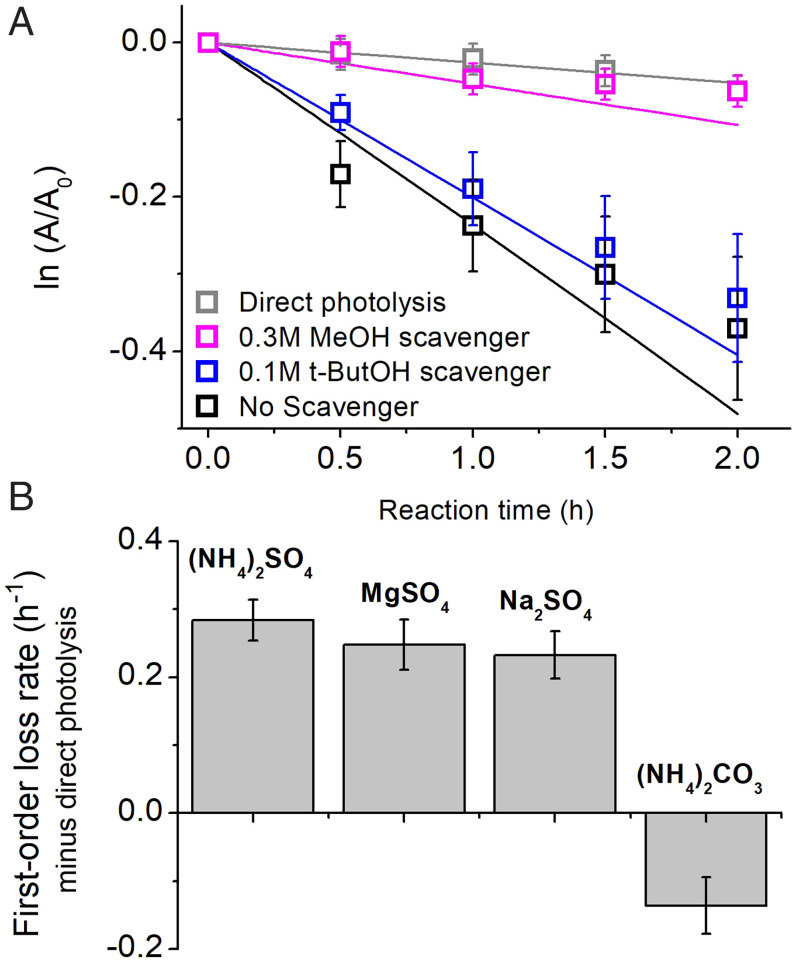
(*A*) Radical scavenging of nitroaromatic + AS + hv reactions using MeOH to scavenge SO_4_ and OH, and *tert*-butanol (t-ButOH) to scavenge OH; (*B*) Nitroaromatic reactions in 3.7 M solutions of various salts. All organic concentrations are 1 mM. Fits in *A* represent kinetic model simulations.

In order to confirm the involvement of sulfate radicals, we take advantage of the selectivity of SO_4_^•–^ reactions. Radical scavengers like methanol (MeOH) react similarly with OH and SO_4_^•–^, but *tert*-butanol reacts preferentially with OH (*SI Appendix*, Table S4, and references therein). For 1 mM the nitrophenol p-NP, we estimate that 0.1 M *tert*-butanol scavenges 92% of OH and 12% of SO_4_^•–^, but 0.3 M MeOH scavenges 98% of OH and 82% SO_4_^•–^. We observe using high-pressure liquid chromatography–high-resolution mass spectrometry (HRMS-HPLC) (and reproduce with a model, Fig. 2*A* solid lines) that MeOH effectively shuts down the reaction in the irradiated AS + organic solutions, while *tert*-butanol only minimally reduces the reaction rates, consistent with the expectation that *tert*-butanol scavenges a minor portion of SO_4_^•–^. Repeated scavenger experiments with another organic compound confirm the results (*SI Appendix*, Fig. S6). These observations do not preclude involvement of OH radicals at some point in the reactions, as sulfate radicals can form OH in water (5); however, the model predicts low OH production from this pathway. We can conclude that OH does not initiate the formation of SO_4_^•–^.

We further demonstrate that it is the anion, not the cation, that is involved in reactions by using different salts. All sulfate salts (AS, MgSO_4_, Na_2_SO_4_) promoted the degradation of the nitroaromatic compound under investigation with similar first-order rate coefficients within uncertainty (Fig. 2*B*). In contrast, the photolysis of ammonium carbonate ((NH_4_)_2_CO_3_) did not promote degradation of the nitroaromatic compound, but rather suppresses its direct photolysis, possibly due to quenching aromatic excited states.

**Fig. 3.**
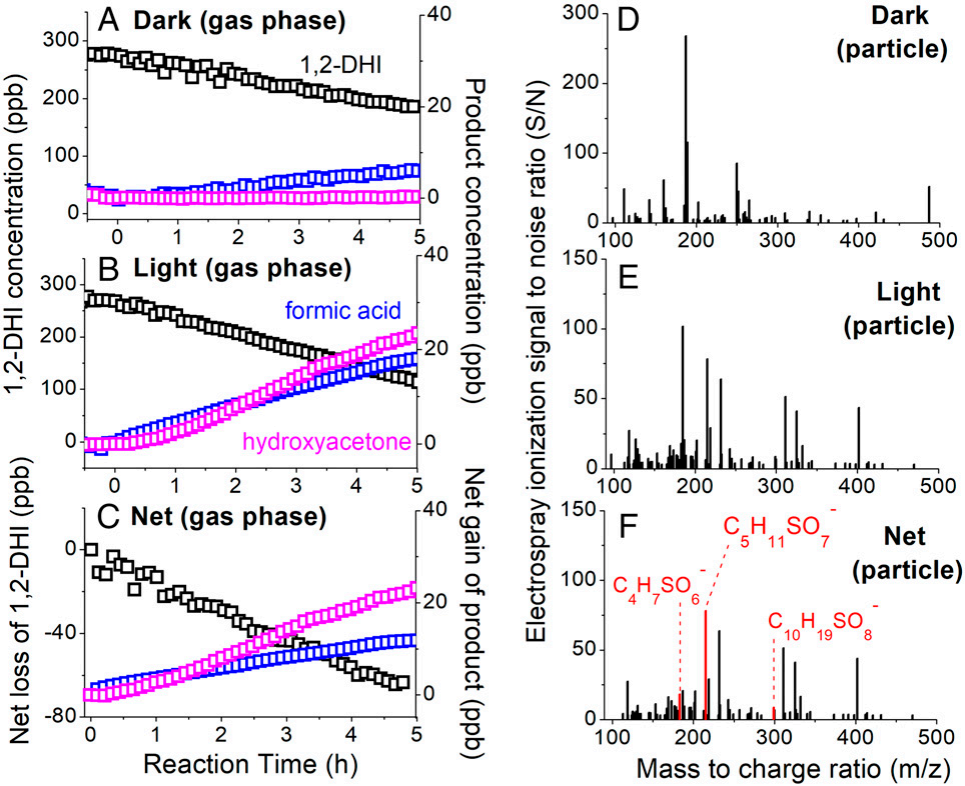
Uptake of 1,2-DHI onto hydrated AS particles in an atmospheric chamber in the absence of OH precursors. Gas phase species from the dark 1,2-DHI + AS (*A*), UV light irradiated 1,2-DHI + AS + hv (*B*), and net (*C* = *B*–*A*, light minus dark) reaction show that 1,2-DHI is lost quicker and decomposition products such as formic acid and hydroxyacetone are formed in the irradiated experiment. Results from a control experiment where an empty chamber was irradiated has been subtracted from the data in (*B*). Associated particle phase organic composition for the dark (*D*), irradiated (*E*), and net (*F*) experiments show that a significant fraction of oxidized products, including the organosulfates highlighted in red, are formed in the particle phase. Particle composition from a dark AS experiment (without 1,2-DHI) have been subtracted from *D* and *E*. Formation pathways from 1,2-DHI + SO_4_^•–^ to notable organosulfates are shown in *SI Appendix*, Fig. S9.

Furthermore, we synthesized an atmospherically important alkene (23), 1,2-DHI, to be used as a radical trap for SO_4_^•–^ in both the bulk aqueous phase and in the liquid water of suspended and hydrated aerosol particles in an atmospheric chamber. Although the addition of SO_4_^•–^ to C = C double bonds to form organosulfates is thought to be minor compared to fragmentation induced by electron transfer or other mechanisms (7), organosulfate formation verifies the formation of SO_4_^•–^ radicals in particle water. We show that, compared to the dark 1,2-DHI + AS chamber experiment (Fig. 3*A*), the loss of the 5-carbon 1,2-DHI and the formation of 1–3 carbon products is substantially higher in the irradiated 1,2-DHI + AS + light experiment, suggesting that reactivity occurs in the irradiated particle. In the dark, the 1,2-DHI is lost to the humid Teflon chamber walls and/or the AS seed particles via equilibrium partitioning of vapors (24). 1,2-DHI is lost to hydrated AS particles at a net rate of ∼14 ppb per hour when irradiated (Fig. 3*C*), producing a substantial amount of formic acid and hydroxyacetone (Fig. 3*C*).

Hydroxyacetone is formed significantly in the 1,2-DHI + AS + light experiment, but not in the dark control (1,2-DHI + AS) nor the light control (empty chamber + light) experiments (*SI Appendix*, Fig. S7*A*). It is also a major product of 1,2-DHI + OH (23) in the gas phase. Acetic acid and glycolaldehyde were significantly observed as well (*SI Appendix*, Fig. S7*C*); however, we did not differentiate between these structural isomers in this work. HRMS analysis of the particles collected onto filters shows that the difference between the light (Fig. 3*E*) and dark (Fig. 3*D*) experiments is a net formation of the isoprene trihydroxy organosulfate (C_5_H_11_SO_7_^–^) and related compounds (Fig. 3*F*), which are absent in the dark experiment. Application of tandem mass spectrometry to the C_5_H_11_SO_7_^–^ precursor ion (*SI Appendix*, Fig. S8) demonstrates that this ion is a covalently-bonded organosulfur species; it produces the HSO_4_^–^ product ion upon collision-induced dissociation.

Approximately 40–90% of organic peaks observed in negative mode electrospray ionization from the 1,2-DHI + AS + hv reaction in the aqueous bulk phase and aerosol phase can be assigned as organosulfates (*SI Appendix*, Tables S5–S7), which confirms that AS solutions irradiated with UVA and UVB radiation (*SI Appendix*, Fig. S1) form SO_4_^•–^ radicals. The proposed product formation mechanisms (*SI Appendix*, Fig. S9) are consistent with those reported for aqueous SO_4_^•–^ reaction with other alkenes (7).

### Concentration Effects.

**Fig. 4.**
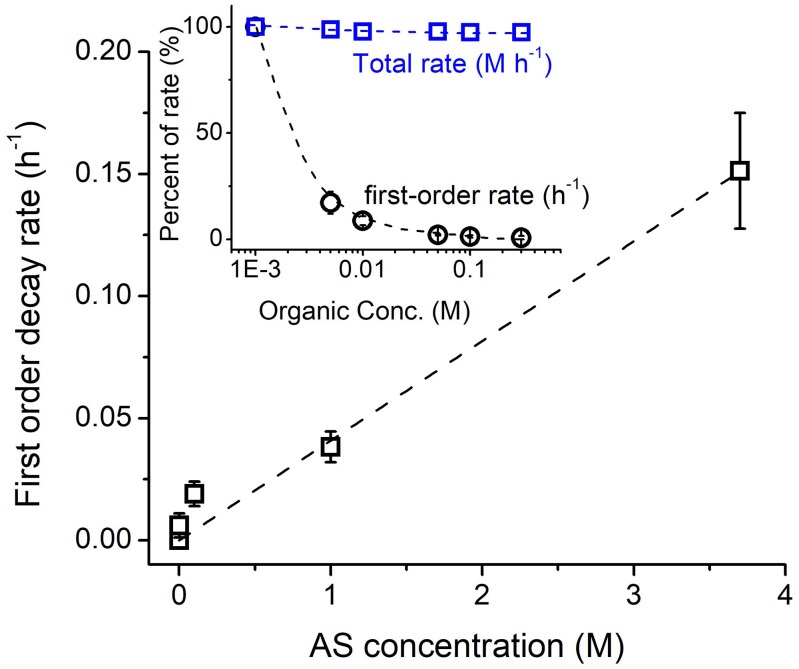
Dependence of the photolytic SO_4_^•–^ reaction on AS concentration and organic concentration (insert). The AS concentration dependence is measured using a nitroaromatic compound (p-NP). The concentration dependence in the insert is modeled using both aromatic and aliphatic systems.

Using p-NP as a model organic, its photochemical decay increases fairly linearly with the ionic strength of the irradiated AS solution (Fig. 4, raw data in *SI Appendix*, Fig. S10). The maximum ionic strength investigated in this work is ∼4 M, which corresponds to a fully deliquesced AS particle at a RH of 85% (3). Higher ionic strengths cannot be tested in this manner as they are unstable and will interfere with measurements. In the atmosphere, RH lower than 85% but higher than the efflorescence point of AS [∼35% (25)] is common, which will further increase the ionic strength of sulfate in aerosol water and may accelerate photochemical sulfate formation.

Organic concentrations also play a role in understanding AS-induced photo-kinetics. Although organic molar concentrations in particle liquid water are not well constrained due to factors such as solubility, molecular size, surface activity, volatility, and phase, they are estimated to be in the millimolar range (26, 27). The pseudo first-order decay (*k*_obs_ = *k*_org+SO4_ × [SO_4_^•–^]_ss_) for both aromatic and aliphatic systems can be modeled to decrease sharply with increasing organic concentration (Fig. 4 insert, black), while the total rate (R = *k*_org+SO4_ × [SO_4_^•–^]_ss_ × [Org]) remains fairly constant (Fig. 4 insert, blue). This is because the lower *k*_obs_ at increasing organic concentration is applied to a higher organic concentration for a fairly constant total rate, i.e., the absolute amount of organics that reacts with SO_4_^•–^ is modeled to remain nearly the same. Extrapolating to atmospheric reactions using the noon-time experiment of p-NP (*SI Appendix*, Fig. S4), we can estimate that the SO_4_^•–^ source described here can oxidize 50–370 μM/h of the tested organics on fully deliquesced aerosols in the summer daytime (*SI Appendix*, Table S8) and potentially more at RH conditions < 85%. This compares reasonably well to presumed OH oxidation rates of ∼270–720 μM/h under similar conditions (*SI Appendix*, Section S1). Furthermore, in heterogeneous reactions such as for 1,2-DHI (Fig. 3), organic mass is reacted from the gas phase and mostly repartitions to the gas phase due to extensive fragmentation caused by SO_4_^•–^; thus, organic reactants need not be situated in the particle phase in order to degrade.

### pH Dependence.

**Fig. 5.**
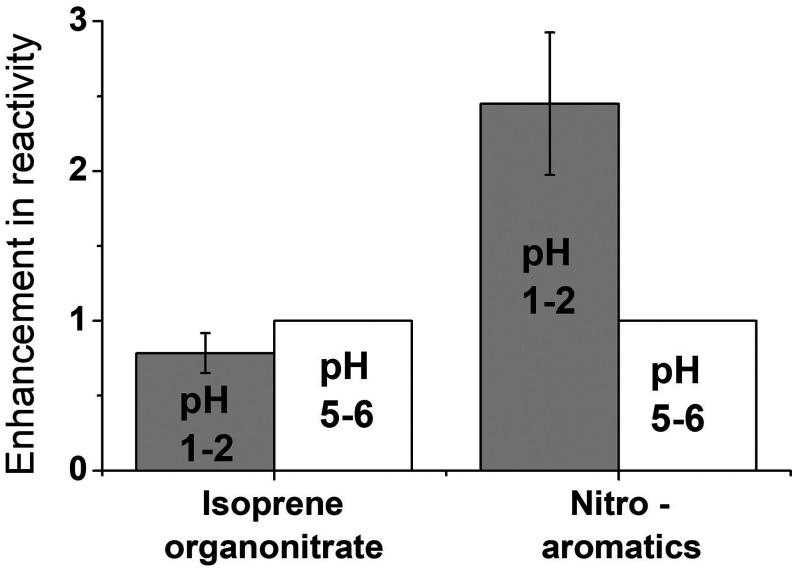
Differences in reactivity in irradiated 3.7 M AS + organic solution at two pH ranges relevant to atmospheric aerosols. The primary organic dinitrate of isoprene (2,3-dihydroxy-1,4-dinitro-isoprene) is not pH-sensitive, while the three nitroaromatic compounds studied have pH-sensitive structures. All reactivities (first-order decays) are normalized to values at pH 5 and direct photolysis rates have been subtracted. Uncertainties are 1 SD in the nitroaromatic data and analytical precision in the isoprene nitrate data.

The pH of liquid water in sulfate aerosol is typically in the range of 1–5 (28). In this pH range, the distribution of sulfur anion species in solution dramatically shifts. The dissociation equilibrium of HSO_4_^–^ ⟷ SO_4_^2–^ + H^+^ has a pK_a_ of ∼2; thus, S(VI) is mostly in the form of HSO_4_^–^ in the AS solutions at pH 1–2, compared to almost entirely in the form of SO_4_^2–^ at pH 5–6. Given that some organics are pH-sensitive themselves, we performed experiments on both pH-insensitive (DHDN) and pH-sensitive (nitroaromatics) model systems. Fig. 5 shows that the reactivity of DHDN in photolyzed AS solution at pH 1–2 decreased ∼20% compared to pH 5–6, which is within analytical uncertainty. However, the reactivity of AS + hv reactions increased by a factor of 2–3 for the three nitroaromatic systems investigated at the lower pH range, significantly higher than uncertainties. Comparatively, direct photolysis rates of the nitroaromatics and isoprene dinitrates are generally not different at different pH (*SI Appendix*, Table S1).

These data suggest that both HSO_4_^–^ and SO_4_^2–^ are sulfur radical sources under the irradiated conditions of this work. The significant enhancement in nitroaromatic degradation at pH 1–2 (Fig. 4) suggests that these aromatic systems may synergistically participate in propagating SO_4_^•–^ or other radicals, consistent with reactions proposed for bisphenol A (22). These data suggest that the more acidic (pH 1–2) aerosol liquid water in the Southeast United States (29) and the less acidic (pH ∼4) aerosol water in agricultural regions in California’s Central Valley (30) may both promote sulfur radical chemistry from aqueous sulfate-containing organic aerosols.

### Effects of Oxygen.

**Fig. 6.**
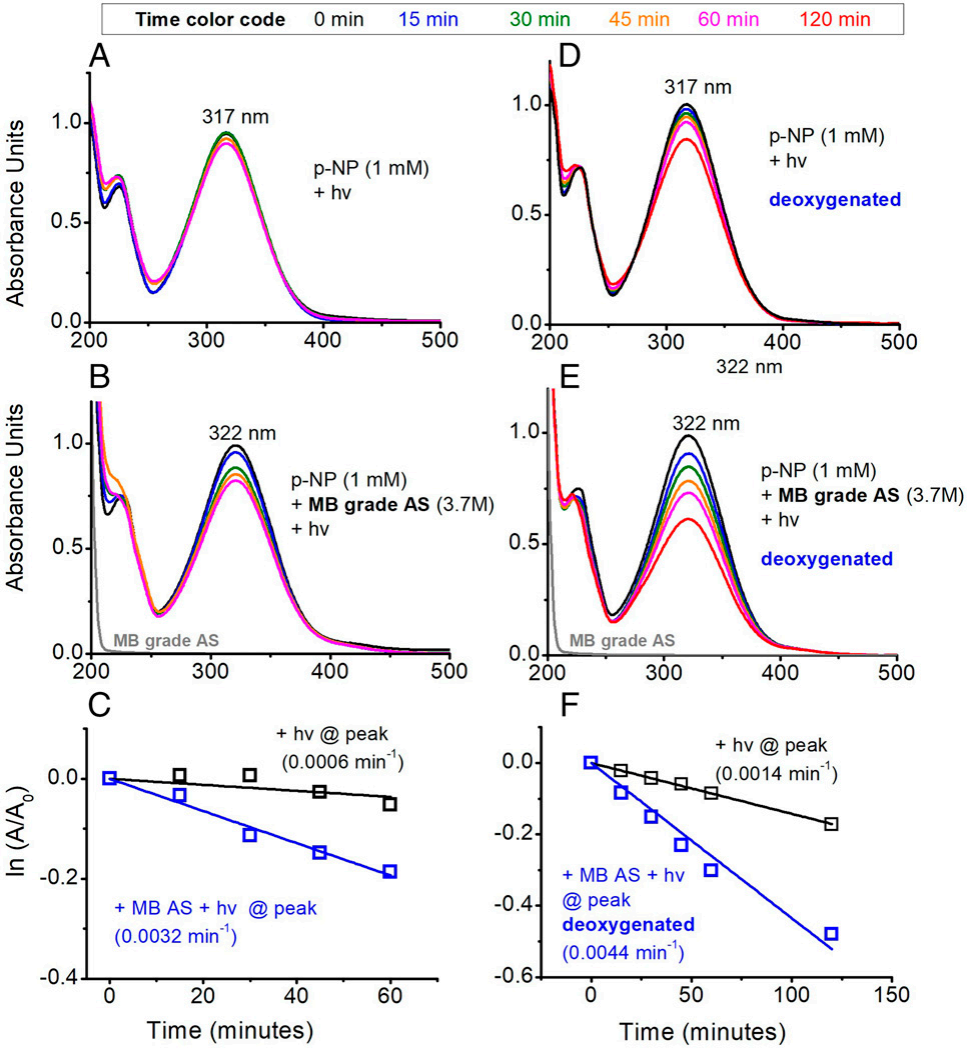
Differences in reactivity of irradiated solutions of p-NP under (*A*–*C*) an air atmosphere compared to (*D*–*F*) an inert nitrogen atmosphere using deoxygenated aqueous solutions. MB grade AS was used for experiments, which did not have observable chromophoric and trace metals impurities (*SI Appendix*, Table S2).

The role of reactive oxygen species were examined using deoxygenated experiments under inert atmosphere using the p-NP reagent (Fig. 6). Lack of oxygen in the reaction accelerates both direct photolysis and the AS-mediated photoreaction; this may be related to oxygen quenching of the p-NP triplet (31). In both cases, the photochemistry is highly enhanced in the presence of the sulfate salt compared to the direct photolysis. Moreover the nitrophenol-AS mixture (pH ∼5.8) reproducibly exhibits a red shift compared to the absorbance spectrum of the pure nitrophenol in water (pH ∼4.8, *SI Appendix*, Fig. S4 and Fig. 6), a phenomenon that cannot be attributed to pH changes (32) but may suggest organic-inorganic interactions. The deoxygenated experiment results further confirm that reactive oxygen species are not driving the reaction.

## Discussion

### Potential Mechanisms for Sulfate Radical Formation.

Aqueous sulfate has previously been shown to form radicals under irradiation at highly energetic wavelengths [185 nm (33)]; however, it has not been thought to undergo the same radical-forming reaction under tropospheric radiation (>300 nm). It is likely that high ionic strengths of sulfate ions in aerosols promote photochemical reaction in the troposphere, whether due to low hydration numbers under environmental conditions (34) that may reduce energy needed for photoreaction or other reasons. For example, the UV energy that is needed to eject an electron from SO_4_^2−^(H_2_O)_n_ to produce SO_4_^•–^ (H_2_O)_n_ is proportional to the number of waters in its hydration shell, and electron detachment is thought to occur spontaneously at 3 waters (35). A fully-solvated sulfate anion in dilute solution contains ∼16 waters in its first solvation shell (36). For SO_4_^2−^(H_2_O)_n_ in aqueous solution, the effective potential barrier to electron detachment ∼9 eV or ∼140 nm for aqueous sulfate (37). Sulfate in particle liquid water, however, is thought to be in a metastable state. It has been shown using Raman spectroscopy of NaNO_3_ bulk aqueous solutions that the number of waters in the hydration shell of NO_3_^−^ decreases from 12 to 4 when increasing concentration from 0.1 M to 4 M (38). Extending this observation to sulfate ions, the effective potential barrier to electron detachment of SO_4_^2−^(H_2_O)_4_ can be estimated to decrease by a factor of three compared to dilute solutions (39).

It is also possible that organic triplets (e.g., from C = O or C = C) produced by irradiation of organic reagents themselves may be quenched with sulfate and bisulfate ions to generate sulfur radicals. This has been shown for SO_3_^2−^ and carbonyl triplets, which can generate high quantum yields of SO_3_^•–^ (40). The radical quantum yield varies linearity with ionic strength of the anion for some anion-organic systems, but not others (31). It may be possible an analogous reaction occurs for SO_4_^2−^ or HSO_4_^−^; this has not yet been shown but may be considered in light of the phenomena described here. Lastly, it is possible that the unique interfacial region of particles plays a role in the reaction during the chamber experiments in this work and in tropospheric aerosols. Air-water interfaces have been demonstrated to promote redox chemistry spontaneously on AS particles (41) and water microdroplets (42, 43). The reaction under study will require further characterization, but clearly can degrade organic compounds using low light energies available on a hazy day (Fig. 1*C*) or near sunset (*SI Appendix*, Fig. S4).

Alternatively, it has been suggested that SO_4_^2−^ is reduced to SO_3_^2−^ in water in the presence of phenolic compounds, which can then form SO_3_^•−^ through irradiation and eventually SO_4_^•–^ in an autoxidation mechanism (22). We can likely rule out this mechanism as photolysis of sulfite and bisulfite requires high light energy [e.g., <260 nm (44)] and phenolic compounds were not present in many of our experiments. Furthermore, neither our mass spectrometry analysis, nor that of Galloway et al. (11), provided evidence of sulfite addition to carbonyls. Recently, reduced sulfur species (such as HS^−^) were shown on AS aerosol surfaces (41); however, we also did not see evidence of thiol (R-SH) products in the reactions under study.

### Source Strength.

SO_4_^•–^ radical concentrations in aerosol water to be estimated in the range of ∼1 × 10^−14^–3 × 10^−14^ M from AS + organic photochemistry on a sunny day at low mM organic concentrations for the nitrophenol system, using the *k*_SO4_ of nitrophenol (45). The same calculation for PA results in ∼1 × 10^−12^ M steady state SO_4_^•–^ using the *k*_SO4_ of cis-PA (4) when the reaction rate is extrapolated to atmospheric light flux. This new SO_4_^•–^ source may add to known sources that are modeled to produce [SO_4_^•–^]_ss_ ∼1 × 10^−14^ M in urban clouds and deliquesced particles (4). Using the SO_4_^•–^ concentration in the nitrophenol system, a bulk radical formation rate of ∼2 × 10^−8^ M s^−1^ at a 3.7 M AS strength can be roughly extracted. This calculation might not be extrapolated to all organic-sulfate systems. This estimated SO_4_^•–^ radical production rate compares reasonably well with the in-situ OH formation rate that has been measured on bulk aerosol particles (∼1 × 10^−9^ M s^−1^) or for marine aerosols (1–10 × 10^−8^ M s^−1^) (46, 47). While there is not enough information at this point to fully confirm molecular mechanisms, the source of SO_4_^•–^ radicals reported here appears to be significant in strength and should be considered in both models and in the interpretation of experimental results.

### Impact.

Cloud droplets, fog droplets, and aerosol particles interconvert in the highly dynamic processes of the atmosphere. Aerosol particles nucleate clouds and fog, and then these aqueous droplets evaporate (48). This process may happen multiple times per hour over a particle’s atmospheric lifetime depending on environmental conditions (49). Thus, the SO_4_^•–^ production described here may occur on aerosol water primarily but impact larger droplets during different stages of aerosol-cloud interactions.

A new SO_4_^•–^ reaction involving sulfate ions may affect our understanding of the global sulfur cycle that helps regulate climate. This is due to the fact that SO_4_^•–^ is an intermediate in the aqueous oxidation of SO_2_, and an oxidant for marine organics in the dimethylsulfide (DMS) oxidation chain (50). Thus, photochemical sources of SO_4_^•–^ may originate from both fossil fuel SO_2_ and marine sulfate aerosols. Losses of SO_4_^•–^ due to a reaction with organics in the S(IV) oxidation chain strongly inhibit SO_2_ oxidation, and thus gains of SO_4_^•–^ from photolytic reaction could also alter SO_2_ autoxidation. Over the oceans, increased SO_4_^•–^ may enhance the degradation of dimethyl sulfoxide (DMSO), an oxidized form of DMS, given that the rate coefficient for DMSO + SO_4_^•–^ is comparable to that of DMSO + OH (50). Other marine sulfur species may also be affected, although kinetic data are not fully available to immediately discern these impacts.

While the SO_4_^•–^ reaction with organics marks the end of the sulfur oxidation chain, it begins the organic oxidation chain. Thus, another important impact of this new SO_4_^•–^ source may be in altering the lifetime of the organics in atmospheric particles. Particle mass may be lost as SO_4_^•–^ reactions tend to fragment aliphatic species, but in some cases may increase if SO_4_^•–^ adds to double bonds, epoxides, or draws in mass from the gas phase. It has been noted that stronger sinks for particle organics are needed to resolve model-measurement discrepancies in the organic aerosol burden (51), and this work provides an example of a previously unknown sink. However, the chemical fate of particle-phase organics is poorly understood, and thus, kinetic measurements of SO_4_^•–^ with a variety of atmospherically relevant organics would be needed before these reactions can be implemented into atmospheric models.

Notably, the SO_4_^•–^ + organic reactions will also modify aerosol composition by producing surface-active species such as organic acids and organosulfates. In particular, we showed that 1,2-DHI forms the isoprene trihydroxy organosulfate when reacting with SO_4_^•–^, which is one of the single most abundant organosulfate species on fine particles and has been exclusively attributed to a dark ring opening of the isoprene epoxide (52). Knowledge of organosulfate formation and degradation mechanisms are currently lacking (8), which may have led to underestimations of organosulfates in modeling studies. The conversion of alkenes, aromatics, and other species to organosulfates through SO_4_^•–^ aging may suppress the aerosol surface tension in the process of nucleating clouds (53), which then modify that aerosol’s indirect effect on climate. Furthermore, given that formic acid is a prominent product of the SO_4_^•–^ oxidation reactions in aerosol water (Fig. 3), volatile compound production from unaccounted SO_4_^•–^ reactions in particle water may help reconcile the “missing” formic acid in the atmosphere (54). However, a detailed understanding of the magnitude and sign of potential outcomes will require further research into radical quantum yields and related physicochemical constants. Improved representation of SO_4_^•–^ sources and reactions in models may aid in simulations of atmospheric composition in future studies.

Production of SO_4_^•–^ from high ionic strength AS solutions or wet particles mixed with organics may also help explain some laboratory observations in the literature that have unknown mechanisms, e.g., photochemical organosulfate formation in the absence of radical oxidant sources (10, 11) or faster organic growth via reactive uptake of aldehydes on irradiated AS particles compared to dark (55). A modeling study of glyoxal uptake on wet sulfate aerosol by Sumner et al. (56) concluded that a major light-dependent mechanism is currently not accounted for by known chemistry. Furthermore, this work can provide additional insights to previous observations where other chemistry is simultaneously active. For example, laboratory studies of secondary organic aerosols (SOA) formation or aging from different chemical systems have been found to be substantially different when performed on dry compared to hydrated AS seeds where high ionic strength sulfate ions are present (57–61), sometimes without a clear reason (62). A gain in SOA mass and higher degree of oxidation in humid systems where SO_4_^•–^ can add [e.g., epoxydiols (57, 63) and aromatics (59, 60)] and a decrease in SOA mass found in humid alpha-lactone system where SO_4_^•–^ can decarboxylate the lactone (61) or humid alpha-pinene SOA systems dominated by saturated aliphatic compounds (63, 64) are consistent with the chemistry discussed here. These effects may occur concurrently with known mechanisms such as nucleophilic addition of sulfate ions. Photolytic aging experiments of organics, thus, may not be probing only organic direct photolysis when they are performed on wet sulfate aerosols. Future atmospheric chamber experiments should consider SO_4_^•–^ reactions in the interpretation of results when using hydrated sulfate seed particles in photochemical experiments, even if OH is the intended radical source.

## Materials and Methods

### Materials.

Organic reagents used in this study are shown in *SI Appendix*, Fig. S11. Erythritol (99%), EDTA (>99%), and magnesium sulfate hexahydrate (≥99%) were purchased from Fisher Scientific. D_2_O (99.8 atom% D), CDCl_3_ (99.8 atom% D), MeOH (≥99%), acetonitrile (MeCN) (≥99%), cyclohexane (≥99%), PA (98%), AS (≥99%, RG and MB grade), ammonium carbonate (≥99%), sodium sulfate (≥99%), DNPH (97%), and 50 wt.% hydrogen peroxide (H_2_O_2_) in water were obtained from Sigma Aldrich. 4-nitrophenol (p*-*NP) (99%) and *tert-*butanol (>99%) were purchased from Alfa Aesar. DNPH was recrystallized prior to use, and its formic acid derivative (FADNPH) was synthesized via a previously reported procedure (65). 2-methylbut-3-ene-1,2-diol and 2,3-dihydroxy-2-methylbutane-1,4-dinitrate were each synthesized according to previous reports (66). All other purchased chemicals were used without further purification. Unless otherwise indicated, experiments were performed using RG sulfate salts. Ultrapure H_2_O was obtained from a Milli-Q purification system (Millipore Sigma, 18 MΩ).

### Characterization Methods.

#### HPLC-HRMS.

HPLC-HRMS was used to quantify p-NP, pinonic acid, THB, and DHDN in bulk aqueous photochemical experiments, representative chromatograms shown in *SI Appendix*, Figs. S12 and S13. Analyses were performed on an Agilent 1100 HPLC coupled to a linear-trap-quadrupole (LTQ-XL) Orbitrap mass spectrometer (Thermo Corp., Waltham MA) operating at a mass resolving power of 60,000 m/Δm at *m/z* 400. Separation of DHDN and polyols was performed isocratically on a Shodex Asahipak NH2P-40 2D column (2 × 150 mm, 4 μm, 100 Å) at flow rate of 0.3 mL/min, column temperature of 40°C, and eluent mixture 90:10 MeCN and water with 0.05% ammonium formate. Analysis of p-NP and organosulfate products was performed with an Agilent Poroshell EC-C18 column (2.1 × 100 mm, 2.7 μm, 120 Å) at flow rate of 0.27 mL/min, column temperature of 30°C, and eluent mixture 40:60 MeCN and water with 0.1% ammonium formate. For solutions containing ammonium sulfate, a 100 μL aliquot of reaction sample was mixed with 900 μL MeOH. This precipitated out the ammonium sulfate, which was filtered off, and the filtrate was analyzed without further purification. For solutions in pure water, a 100 μL aliquot of reaction sample was diluted with 900 μL MeOH and used without further purification.

#### Direct Infusion HRMS.

Aerosol particles collected from chamber experiments were extracted with 0.5 mL LC-MS grade MeCN (Fisher Optima) and 2 min sonication in a bath sonicator. The extract was then directly introduced into the Orbitrap mass spectrometer (above) at the same tuning specifications but with a mass resolving power of 30,000 m/Δm at *m/z* 400 to improve sensitivity. External mass calibration is performed using commercial ESI calibration mix (Pierce Negative Mode Calibration solution). Calibrant analytes achieve a mass accuracy of <2 ppm after recalibration. The sample mass spectra with signal to noise ratio (S/N) > 3 were processed by subtracting the mass spectra of the control filter extracts, deconvoluted with a quadratic fit model and deisotoped using Decon2LS tools (freeware from PNNL), mass corrected with the external calibration curve, and assigned to molecular formulas using a custom Matlab protocol based on heuristic mass filtering rules (67) and Kendrick Mass (KM) defect analysis (68, 69) with KM base of CH_2_.

#### ^1^H NMR.

Due to the large HOD/H_2_O and NH_4_ signals, 50 mM of 1,2-DHI was mixed with 3.7 M AS in D_2_O to increase the analytical signal. Reactions for NMR analyses were performed by directly irradiating the sample within a 5 mm quartz NMR tube rated for 500 MHz. ^1^H spectra were collected on a 400 MHz Bruker instrument (400 MHz Bruker Avance III HD Nanobay Spectrometer) with water suppression, using an autosampler and analyzed using TOPSPIN. Water suppression was run using the standard WATERSUP parameters. Cyclohexane was used as an internal standard. Cyclohexane in CDCl_3_ (0.8 vol.%) was used as an internal standard; the internal standard mixture was loaded into a glass capillary, flame sealed, and dropped into an NMR tube containing the reaction mixture. The solution was irradiated for 1, 24, and 48 h. Aqueous 1,2-DHI first-order loss rates were extrapolated down to 1 mM by kinetic modeling.

#### Gas chromatography mass spectrometry (GC MS).

Analyses were performed on an Agilent 6890N gas chromatograph coupled to an Agilent 5973N quadrupole mass spectrometer using a silylation procedure previously reported for aqueous alcohols (70). Calibration was performed with pure sample in AS solutions to confirm the linearity of this method using our system.

#### UV-Vis spectroscopy.

UV-vis spectroscopy was used to characterize the absorption of reagents, and for kinetic analyses of some reactions of p-NP (in addition to HPLC-HRMS). Spectra were obtained on a Shimadzu UV-1800 UV Vis spectrometer. Samples were directly inserted into the instrument between irradiation without any alterations. Spectra were collected every 15 min for 1 h. It is noted that UV-Vis determination of kinetics for p-NP taken at the ∼320 nm peak are nearly identical to determinations using HPLC-HRMS (*SI Appendix*, Table S1).

#### Inductively coupled plasma (ICP)-MS.

AS solutions (0.15 M) in ultrapure water were analyzed for trace metals using a Thermo Scientific iCAP RQ ICP-MS operating in KED mode (He atmosphere). The analytical matrix is 2% vol/vol Omnitrace HNO_3_ and 0.5% vol/vol Omnitrace HCl. The instrument was calibrated using a 43-element calibration solution (Inorganic Ventures IV-ICPMS-71A). A 6-element internal standard (IV-ICPMS-71D) was added to samples prior to quantitation. Quality control checks were performed using NIST-1643f and Inorganic Ventures IV-STOCK-50 reference solutions.

#### Chemical ionization mass spectrometry (CIMS).

1,2-DHI, formic acid, and hydroxyacetone were quantified using a custom-built triple-quadrupole CIMS using CF_3_O^−^ as the reagent ion. Details of the instrument and the humidity-dependent calibration methods have been described previously (71, 72). Authentic standards were used to calibrate the CIMS; formic acid and hydroxyacetone were purchased from Sigma Aldrich, and 1,2-DHI was synthesized as previously described (23). Formic acid was detected as its F-transfer ion at *m/z* 65. Hydroxyacetone and 1,2-DHI were detected as clusters with CF_3_O^−^ at *m/z* 159 and *m/z* 187. Calibrated signals in CIMS have a quantification uncertainty of 20–30%.

### Bulk Aqueous Experiments.

Photochemical reactions of bulk solutions (Figs. 1, 2, 4–6) were performed in a photochemical enclosure equipped with a UVB broadband fluorescent light with peak wavelength emission at 310 nm (*SI Appendix*, Fig. S1). For all non-NMR studies prior to irradiation, a 5 mL solution of the organic compound of interest at the desired organic concentration with 3.7 M AS in milli-Q water was prepared. For pH-dependent studies, H_2_SO_4_ was added to the solution until pH 1–2 was achieved. All other determinations were performed at pH 5–6. The pH of solutions was measured with a micro pH electrode (LE422) that was calibrated with commercial pH standards. The aqueous solution was transferred to a 3.5 mL capped quartz cuvette with stopper (Thor Labs). This was placed in the photochemistry chamber to irradiate for the desired reaction time. Aliquots of ∼100 μL volume were taken for the chemical analysis procedure specific to the target organic analyte (GC-MS for erythritol, NMR for 1,2-DHI, HPLC-HRMS for others; see instrument-specific subsections above) using Hamilton gas-tight syringes. Reactions performed using natural sunlight as the irradiation source were prepared in the same way as those using artificial lighting. One control sample (organic in water) and one treatment sample (organic + 3.7 M AS in water) in quartz vials were placed on the roof of Meyer Hall at the UC Davis main campus lying on a reflective aluminum sheet (Anomet Inc.) and exposed to sunlight. Samples were collected over several hours and analyzed. Deoxygenated experiments for the p-NP reaction were performed using water sparged with ultra-high purity nitrogen (N_2_) for 2 h at room temperature. Solutions were then prepared in quartz vials, irradiated inside of a glove bag under pure N_2_ atmosphere, capped, and taken out of the glove bag for UV-Vis analyses at various time intervals. Analytical uncertainties represent 1 SD of data across repeated experiments for the majority of experiments. For the 1,2-DHI experiment, uncertainties also include errors resulting from data extrapolation using a model.

### Chamber Experiments.

The aerosol experiments shown in Fig. 3 were conducted using a 10 m^3^ Teflon atmospheric chamber, temperature controlled to 20°C. Prior to experiments, the chamber was cleaned by humidifying to 80% RH, injecting with excess H_2_O_2_ and irradiated with UV lights for 12 h to remove any deposited organic materials before being flushed with dry, purified air for at least 24 h. For each experiment, the chamber was humidified to ∼80% RH prior to all injections using ultrapure water (18 MΩ, Millipure Milli-Q) at 35°C circulated through a Nafion membrane humidifier while purified air flowed through the humidifier and into the chamber. Temperature and RH were monitored continuously by a membrane probe (Vaisala Inc.) calibrated with saturated salt solutions in the RH range of 11–95%. Seed particles were generated by atomizing 20 mM AS through a wet wall denuder. 1,2-DHI was injected in the gas phase by flowing ultrapure nitrogen gas through a clean glass bulb with a few drops of the neat standard until desired concentration is achieved, as monitored by the CIMS. A scanning mobility particle sizer (SMPS), comprising an electrostatic classifier (TSI 3080) and a condensation particle counter (TSI 3772), was used to determine the particle size distribution and number concentration. Approximately 1,500 μg/m^3^ total particle mass (including water) was used for experiments. Particles were sampled shortly after lights were turned on in the chamber via a 20 LPM flow through a hydrophilic PTFE membrane filter with 0.2 μm pores (Omnipore, Millipore Sigma). Approximately 6 h of collection time was used to obtain sufficient organic signal for the extraction to avoid sample concentration.

### Kinetic Modeling.

We use a kinetic model previously described in Paulot et al. (73) and add the sulfate radical mechanisms and photolytic parameters shown in *SI Appendix*, Table S3, primarily from CAPRAM (4, 74), Chu and Anastasio (21), and Wine et al. (75). The chemistry is initiated with the light flux measured in our laboratory (*SI Appendix*, Fig. S1), as well as concentrations used in experiments. The mechanism also uses reaction parameters from the JPL Chemical Kinetics and Photochemical Data Evaluation (76), and is run on Matlab 2021 (MathWorks, Inc.) using its ordinary differential equation (ODE) solver.

## Supplementary Material

Supplementary File

## Data Availability

Experimental results and aqueous raw data have been deposited in ICARUS and Dryad (https://icarus.ucdavis.edu/experimentset/226; 10.25338/B8X34D) (77, 78).
