# Supplementary material for: Sulfur radical formation from the tropospheric irradiation of aqueous sulfate aerosols
Source: Proc Natl Acad Sci U S A. 2022 Aug 29;119(36):e2202857119. doi: 10.1073/pnas.2202857119 (PMC9457335; doi:10.1073/pnas.2202857119)
Supplement: Supplementary File [file pnas.2202857119.sapp.pdf]

PNAS
www.pnas.org

Supplementary Information for

Sulfur radical formation from the tropospheric irradiation of aqueous sulfate aerosols.

James D. Cope[a,†], Kelvin H. Bates[a,b,†], Lillian N. Tran[a], Karizza A. Abellar[c], Tran B. Nguyen[1,a]

[a] Department of Environmental Toxicology, University of California Davis, Davis, California 95616, United States

[b] Center for the Environment, Harvard University, Cambridge, Massachusetts 02138, United States

[c] Department of Chemistry, University of California Davis, Davis, California 95616, United States

† These authors contributed equally

[1] To whom correspondence should be addressed. E-mail: tbn@ucdavis.edu

This PDF file includes:

Supplementary Text
Figures S1 to S13
Tables S1 to S8
SI References

Supplementary Information Text

S1. Calculation of OH oxidation rate for atmospheric organics. Comparisons with OH chemistry may be done with the caveat that measurements of $[OH]_{ss}$ extrapolated to aerosol water conditions are limited and the discrepancies between measured and modeled values are high (1). For a model compound such as erythritol, we can assume a dissolved organic concentration at 100 mM and $k_{Eryth+OH}$ of 2 x 10^9 M^{-1} s^{-1} (2). Particle water $[OH]_{ss}$ measurements span the range of roughly 4 x10^{-16} M to 1 x10^{-15} M (3-5), which corresponds to an OH oxidation rate of roughly 270 - 720 μM/h, if the OH reacts mainly with organics. Thus, the AS + org + hv reaction as a sink for organics in particle water may be competitive with OH. In particle water with pH < 2, OH will also react with HSO_4^- to produce $SO_4^{\bullet -}$ with a rate constant of 3.5 x 10^5 M^{-1} s^{-1} (6), which may further enhance the organic oxidation potential of $SO_4^{\bullet -}$.

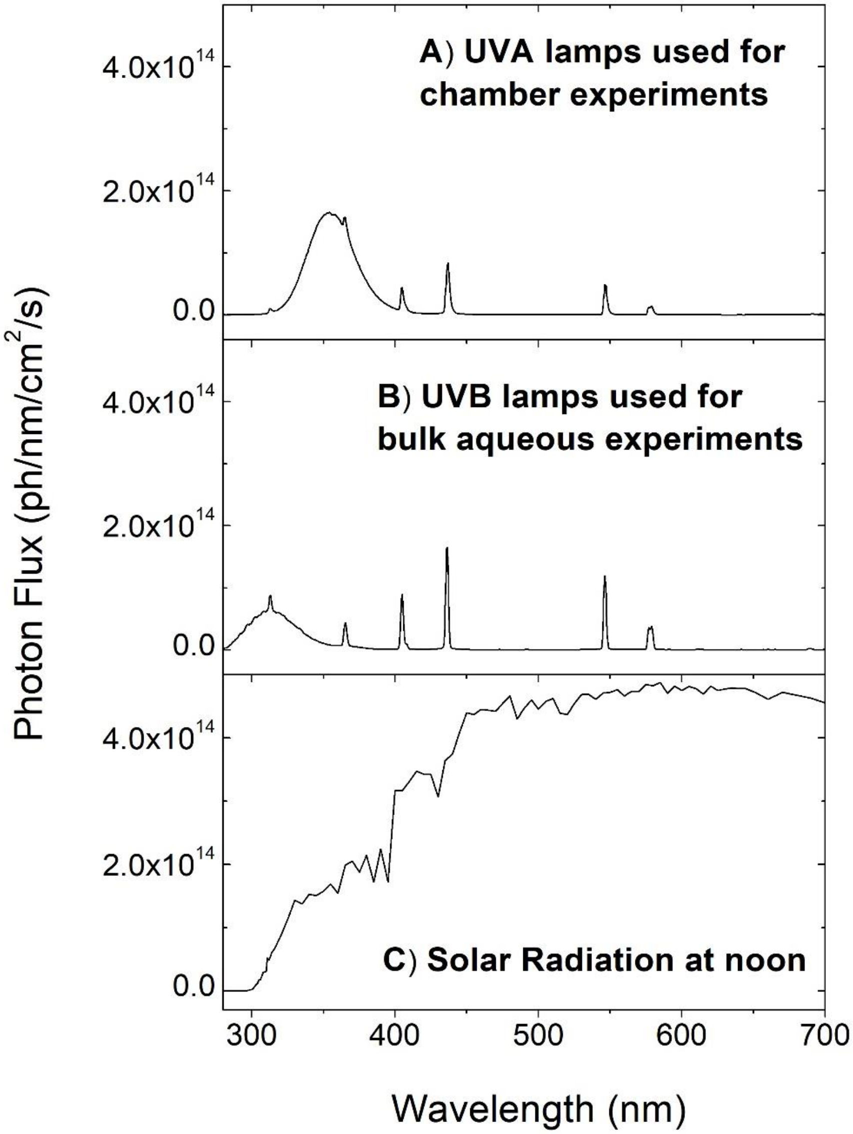

Figure S1. Emission flux of lamps used in A) chamber experiments and B) aqueous experiments, as compared to C) the solar flux at solar zenith angle of 0°C and 300 DU of ozone. The spectral distribution from **A** is obtained from a spectroradiometer (Apogee Instruments) and the flux is obtained by kinetic modeling of a gaseous isoprene + H_2O_2 + hv experiment where all kinetic constants are known (7), isoprene decay is quantitatively constrained by GC-FID data calibrated by isoprene chemical standards, and H_2O_2 data is quantitatively monitored by CF_3O^- CIMS (8). The spectral distribution from **B** is obtained from the same Apogee spectroradiometer and similarly constrained through modeling an aqueous erythritol + H_2O_2 + hv experiment where all aqueous kinetic information is known (2, 9, 10) and erythritol is quantitatively determined from GC-MS following trimethylsilylation (11) – data shown in **Figure 1A**. The flux from **C** is obtained from the TUV model and scaled down slightly to match observed solar actinic flux reported by Kanaya *et al.* (12). Figures are shown on the same scale for ease of comparison.

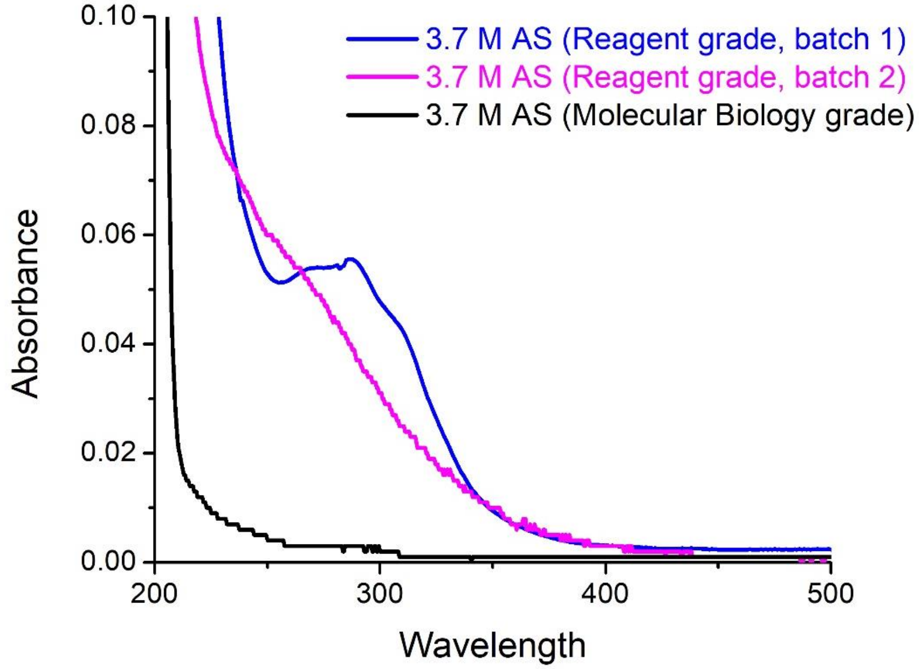

Figure S2. UV-Vis absorbance spectrum of aqueous ammonium sulfate solutions of different purity grades and batches. The Molecular Biology Grade AS does not have detectable absorbing organic impurity. The Reagent Grade AS contains an organic impurity that absorbs in the ~290 nm region with estimated concentration of ~ 10 μM at 3.7 M AS, and mole fraction of ~ 0.0003% assuming a moderate absorbance coefficient of ~ 5000 M^{-1} cm^{-1}.

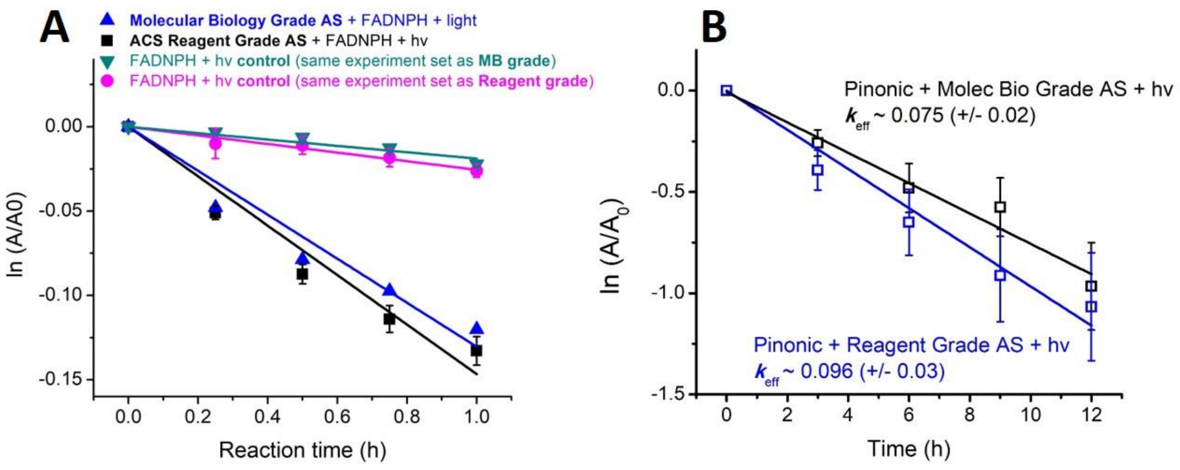

Figure S3. Photochemistry experiments of FADNPH (nitroaromatic) and pinonic acid and with molecular biology (MB) and reagent grade (RG) ammonium sulfate (AS) under irradiation with UVB lamps. Panel **A** data were taken using UV-Vis spectroscopy. Panel **B** data were taken using HPLC-HRMS, with pinonic acid separated from isomers in the analytical column (see **Fig. S13**).

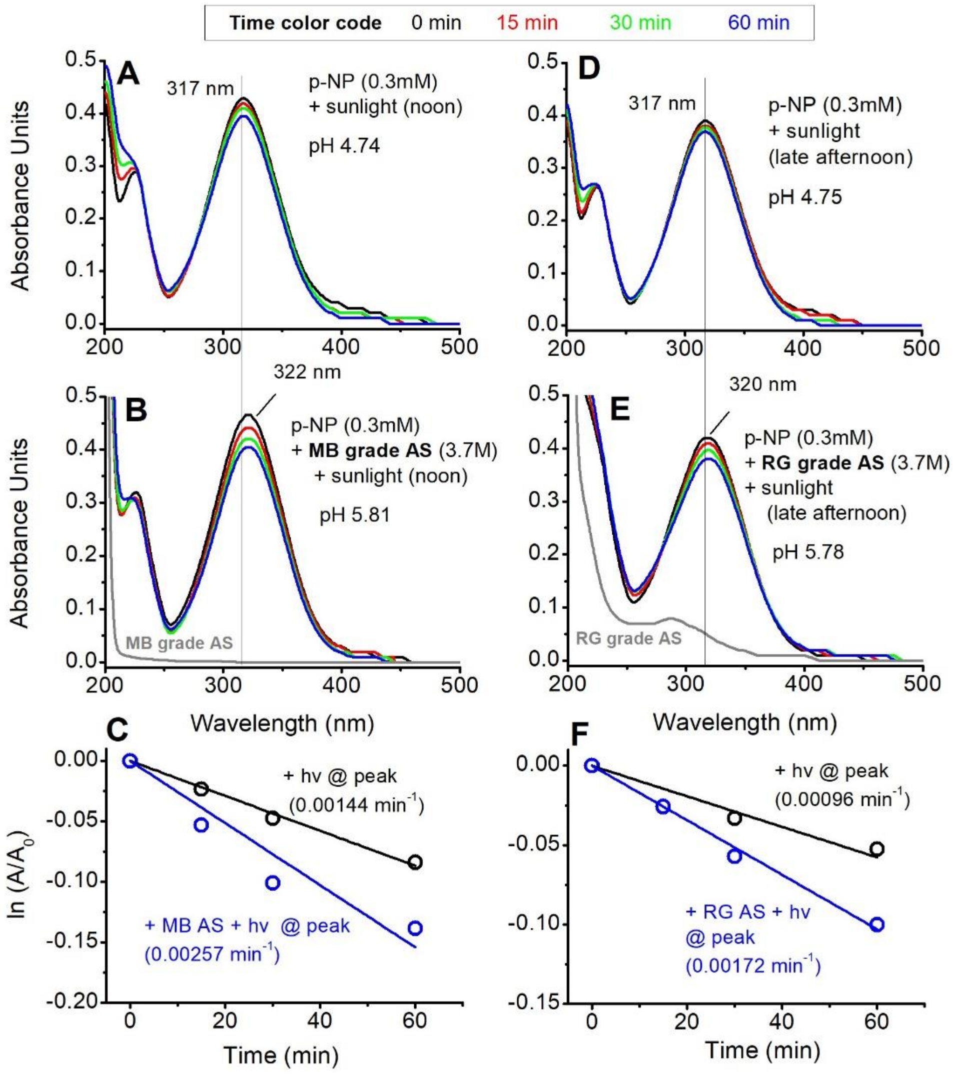

Figure S4. Photochemistry experiments of para-nitrophenol (p-NP) in sunlight at the top of Meyer Hall at UC Davis on a clear summer day. Panels **A** and **B** show the comparison of direct photolysis of p-NP and p-NP + 3.7M molecular biology (MB) grade AS using sunlight at noon. Panels **D** and **E** show the same comparison, but in the late afternoon (3-5pm) using reagent grade (RG) AS. Panels **C** and **F** show the kinetic decay of the MB AS and RG AS induced photochemistry enhancement, respectively.

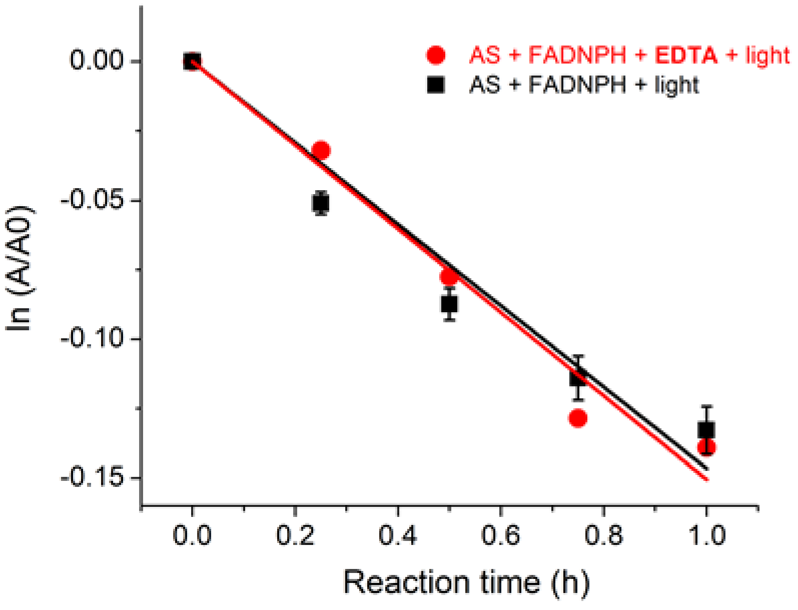

Figure S5. Kinetic determinations of the FADNPH (nitroaromatic) organic reagent with irradiated Reagent Grade AS (3.7 M ionic strength) in the presence and absence of 10 ppm of Ethylenediaminetetraacetate (EDTA) metal chelating agent showed no significant difference in rates of decay. A large excess of EDTA was used to promote chelation of trivalent ferric ions that has lower affinity for EDTA than divalent ions.

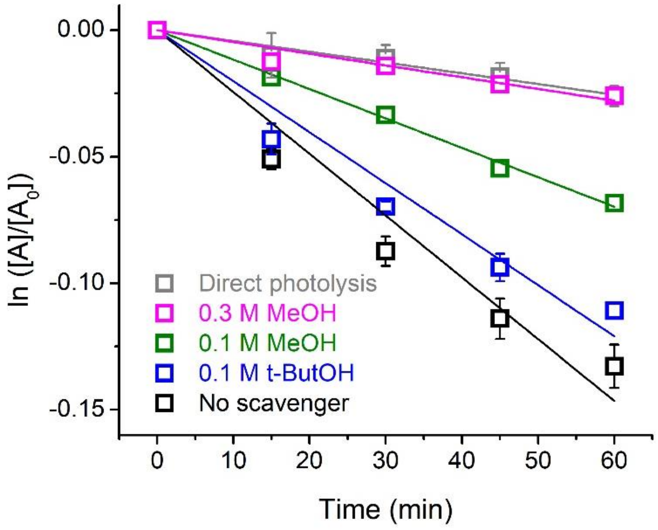

Figure S6. Equivalent radical scavenging experiment shown in **Fig. 2A**, but with FADNPH (nitroaromatic) as the model system. 1 mM organic and 3.7 M AS was used for all experiments except the direct photolysis control (no AS). Kinetics were monitored with UV-Vis spectroscopy.

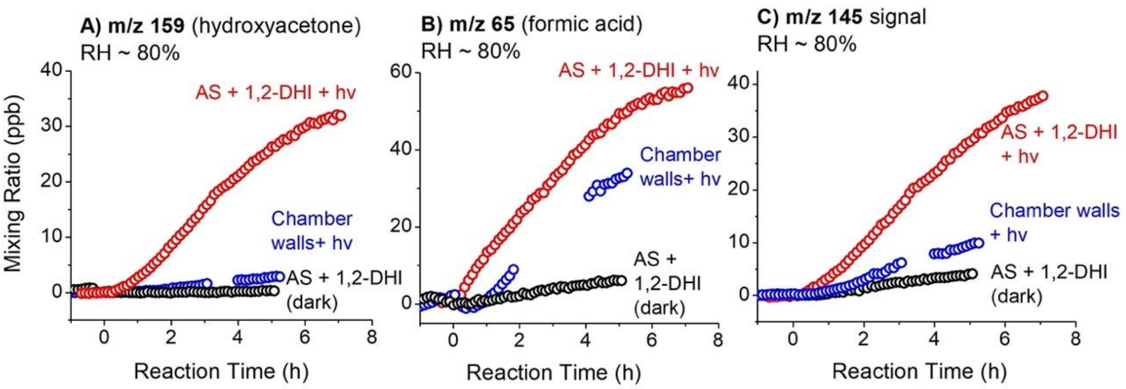

Figure S7. Mixing ratios of oxygenated volatile organic compounds observed by CF_3O^- CIMS in three atmospheric chamber experiments: an irradiated mixture of 1,2-dihydroxyisoprene (1,2-DHI) and hydrated ammonium sulfate (AS) particles (red traces), a dark mixture of the same reactants (black traces), and a control experiment in which the empty chamber was irradiated (blue traces). Data for hydroxyacetone (**A**) and formic acid (**B**) are also shown in **Fig. 3** in the main text. The CIMS signal at *m/z* 145 (**C**) includes contributions from both glycolaldehyde and acetic acid, which we do not attempt to distinguish here. All three experiments were performed at a relative humidity (RH) of ~80% and a temperature of 20 ˚C.

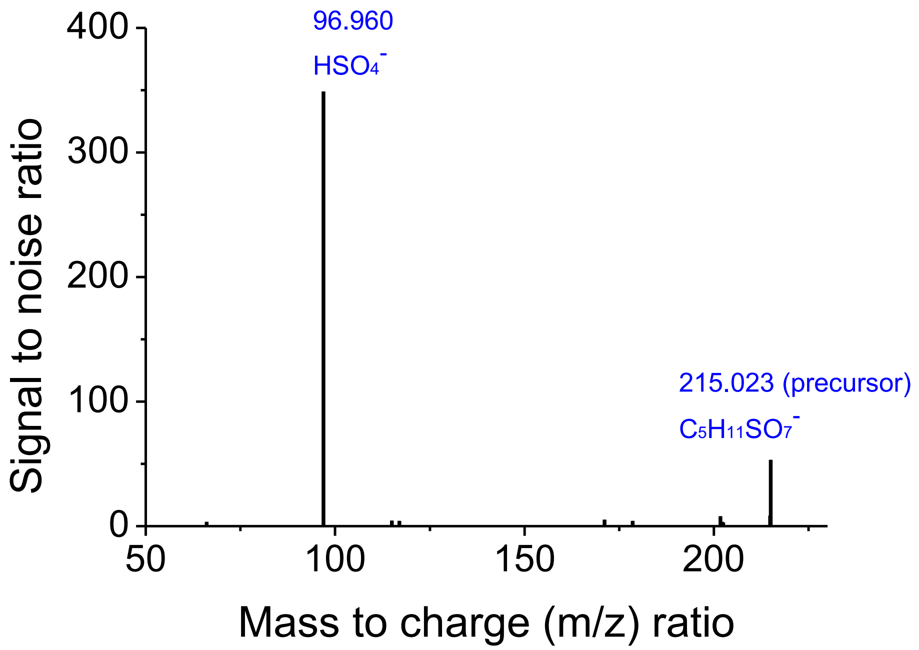

Figure S8. Collision-induced dissociation (CID) tandem mass spectrometry (MS/MS) spectrum of m/z 215.023 at 25 normalized CID energy (0 is no energy applied), which fragmented primarily to HSO_4^- ion.

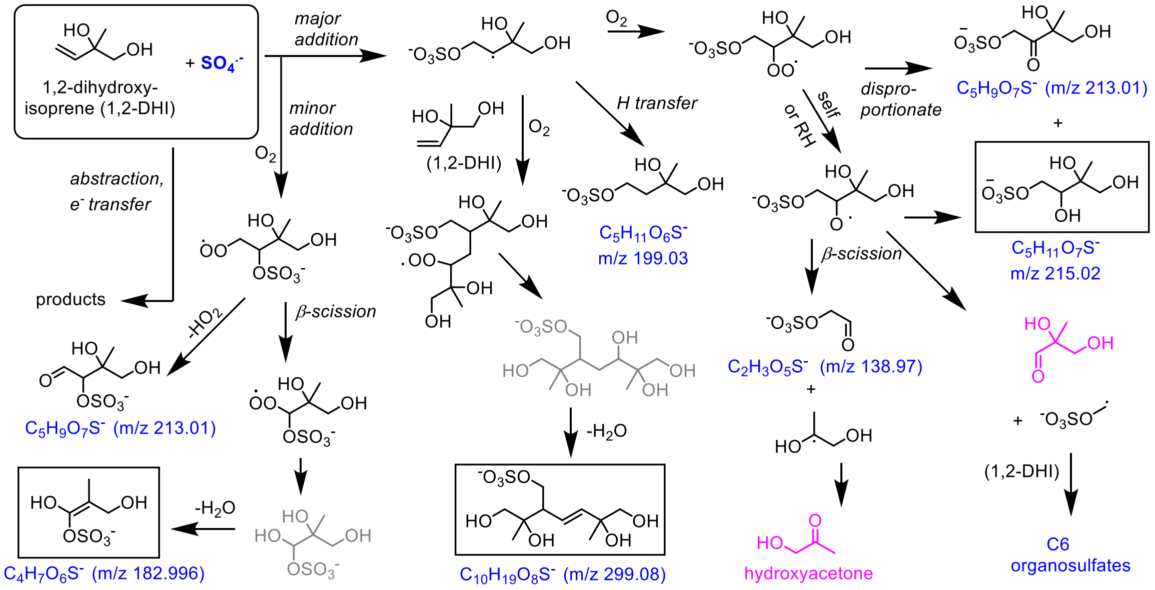

Figure S9. Proposed sulfate radical reaction mechanism with 1,2-DHI to form select organosulfates (blue) and volatile products (magenta). Proposed structures for organosulfates highlighted in **Figure 3** of the main text are shown in boxes. Only the radical addition mechanism is shown here, which is thought to be a minor pathway for reaction, whereas fragmentation-based pathways are thought to dominate the reaction of $SO_4^{\bullet -}$ + alkenes (13). $SO_4^{\bullet -}$ is known to facilitate electron transfer, but in some cases can add to double bonds and/or facilitate elimination reactions (14). Alkyl radicals are expected intermediates when sulfate radicals add to double bonds, in analogy with OH reactions, which will proceed to form peroxy radicals (RO_2) in aerobic water. Atmospheric radical reactions (15, 16) consistent with those recently proposed by Elrod and coworkers (13) can predict nearly all observed organosulfate formation in this work. Dehydrations can occur in the particle water or in the electrospray ionization mechanism.

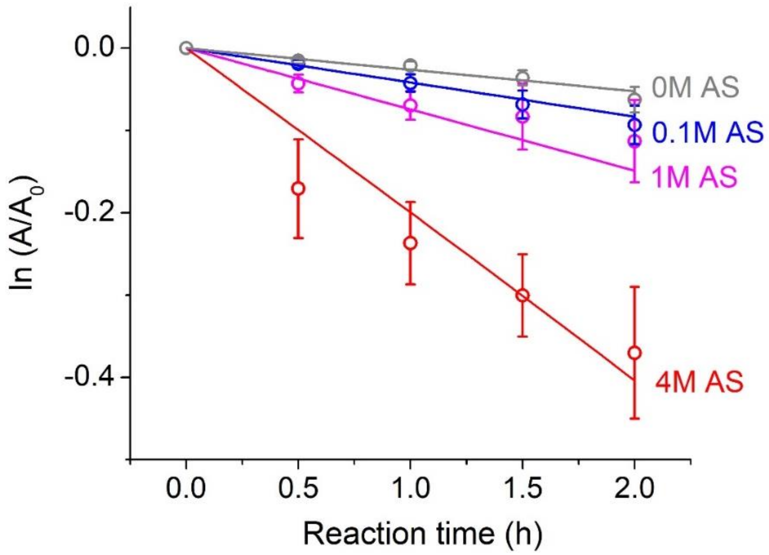

Figure S10. Measured and modeled decays in the photolytic reaction of ammonium sulfate (AS) with p-NP at different ionic strengths of AS. These data are also visualized in **Figure 4** of the main text.

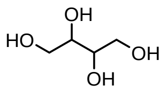

Erythritol
butane-1,2,3,4-tetrol
Molecular Formula: $C_4H_{10}O_4$

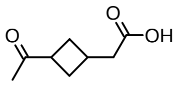

Pinonic Acid (PA)
(3-acetylcyclobutyl)acetic acid
Molecular Formula: $C_8H_{12}O_3$

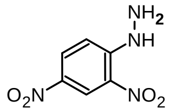

2,4-dinitrophenylhydrazine (DNPH)
(2,4-dinitrophenyl)hydrazine
Molecular Formula: $C_6H_6N_4O_4$

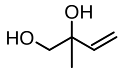

1,2-dihyroxyisoprene (1,2-DHI)
2-methylbut-3-ene-1,2-diol
Molecular Formula: $C_5H_{10}O_2$

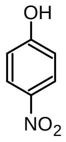

para nitrophenol (p-NP)
4-nitrophenol
Molecular Formula: $C_6H_5NO_3$

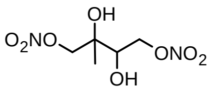

isoprene dihydroxydinitrate (DHDN)
2,3-dihydroxy-2-methylbutane-1,4-diyl dinitrate
Molecular Formula: $C_5H_{10}N_2O_8$

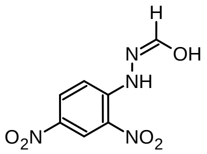

formic acid DNPH (FADNPH)
(2,4-dinitrophenyl)methane-hydrazonic acid
Molecular Formula: $C_7H_6N_4O_5$

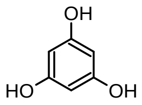

1,3,5-trihydroxybenzene (THB)
benzene-1,3,5-triol
Molecular Formula: $C_6H_6O_3$

Figure S11. Chemical structures, informal names, abbreviations used in the paper, IUPAC names, and molecular formulas for organic reagents.

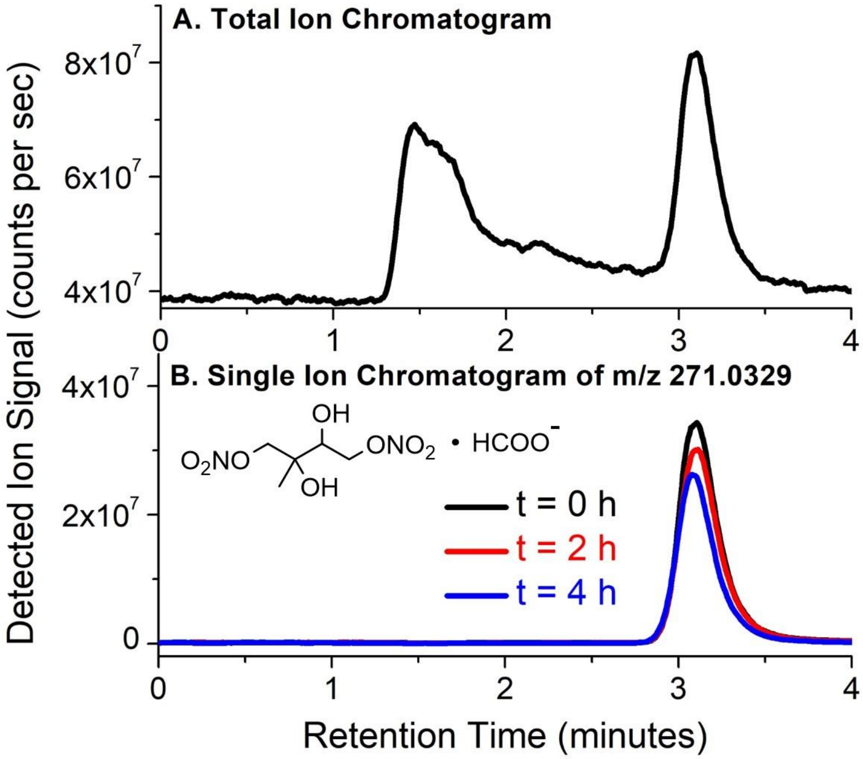

Figure S12. Representative high performance liquid chromatography – high resolution mass spectrometry (HPLC-HRMS) spectra for the kinetic determination of the isoprene dihydroxydinitrate (DHDN, detected as a cluster ion with formate at accurate mass m/z 271.033). The spectrum in **A** shows the total ion chromatogram (TIC) with all detected signals, including any products or reagents, and the spectra in **B** show the single or extracted ion chromatogram of just the dihydroxydinitrate reagent. Integrated peak areas are used to monitor reagent concentration at multiple time points after the start of the UVB irradiation.

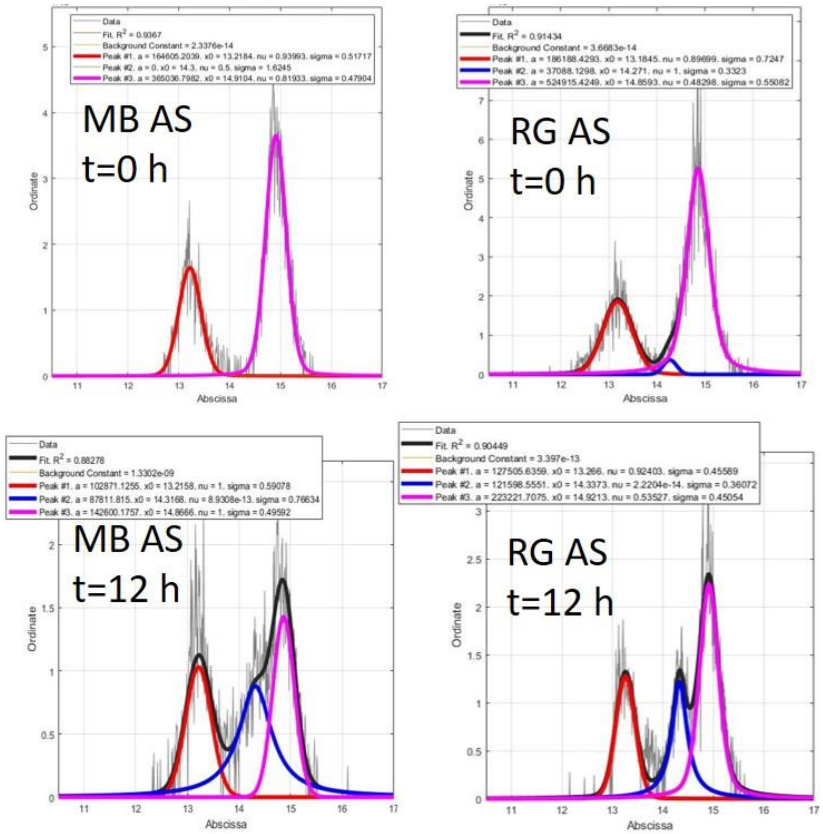

Figure S13. Representative high performance liquid chromatography – high resolution mass spectrometry (HPLC-HRMS) spectra for the kinetic determination of pinonic acid (detected as a deprotonated ion at accurate mass m/z 183.102) photochemistry with molecular biology (MB) and reagent grade (RG) ammonium sulfate (AS) at 3.7M ionic strength prior to irradiation (t=0) and after irradiation for 12 h. A peak in blue grows in that has the same molecular formula as the parent ion, proposed here to be a non-acid compound $C_9H_{14}O$ that clusters with formate, likely arising from sulfate radical induced decarboxylation of the carboxylic acid. The compound in red may be an isomer of pinonic acid; however, it does not decrease in integrated peak area over the course of the experiment.

The deconvolution of peaks was performed in Matlab, using the "Peak fitting to either Voigt or LogNormal line shapes" software package written by Maxim (2022). (https://www.mathworks.com/matlabcentral/fileexchange/52321-peak-fitting-to-either-voigt-or-lognormal-line-shapes), MATLAB Central File Exchange. Retrieved May 18, 2022.

Table S1. Pseudo first order rates observed for experiments in this work. Repeated experiments are shown as average values, with 1-σ uncertainties of 5 – 25% between trials.

#	Organic Compound	[Organic] (mM)	[AS] (M)	Other Reagents	Light	pH	Decay rate (h^{-1})	Method
1	DNPH	1	3.7		dark	5.5	0.01	UV-VIS
1	DNPH	1	3.7		dark	5.5	0.01	UV-VIS
2	DNPH	1	0		hv	5.5	0.20	UV-VIS
3	DNPH	1	3.7		hv	5.5	0.61	UV-VIS
4	DNPH	1	0		hv	2	0.21	UV-VIS
5	DNPH	1	3.7		hv	2	1.13	UV-VIS
6	DNPH	1	1		hv	5.5	0.49	UV-VIS
7	DNPH	1	0	1M $(NH_4)_2CO_3$	hv	5.5	0.07	UV-VIS
8	DNPH	1	0	1M $MgSO_4$	hv	5.5	0.45	UV-VIS
9	DNPH	1	0	1M Na_2SO_4	hv	5.5	0.43	UV-VIS
10	FADNPH	1	0		hv	6	0.03	UV-VIS
11	FADNPH	1	3.7		dark	5.5	0.00	UV-VIS
12	FADNPH	1	0.1		hv	5.5	0.03	UV-VIS
13	FADNPH	1	1		hv	5.5	0.07	UV-VIS
14	FADNPH	1	3.7	RG AS	hv	5.5	0.15	UV-VIS
15	FADNPH	1	3.7		hv	2	0.53	UV-VIS
16	FADNPH	1	0		hv	2	0.07	UV-VIS
17	FADNPH	1	3.7	MB AS	hv	5.5	0.14	UV-VIS
18	FADNPH	1	3.7	RG AS, EDTA	hv	5.5	0.15	UV-VIS
19	FADNPH	1	3.7	0.1M MeOH	hv	5.5	0.07	UV-VIS
20	FADNPH	1	3.7	0.1M tButOH	hv	5.5	0.14	UV-VIS
21	FADNPH	1	1	0.3M MeOH	hv	5.5	0.03	UV-VIS
22	p-NP	1	0		hv	6	0.03	LCMS
23	p-NP	1	0.1		hv	5.5	0.05	LCMS
24	p-NP	1	1		hv	5.5	0.08	LCMS
25	p-NP	1	3.7		hv	5.5	0.20	LCMS
26	p-NP	1	3.7	0.1M tButOH	hv	5.5	0.17	LCMS
27	p-NP	1	3.7	0.3M MeOH	hv	6.5	0.04	LCMS
28	p-NP	1	0		hv	5.5	0.03	UV-Vis
29	p-NP	1	3.7	MB AS	hv	5.5	0.19	UV-Vis
30	p-NP	1	0	DeOxy	hv	5.5	0.08	UV-Vis
31	p-NP	1	3.7	MB AS, DeOxy	hv	5.5	0.26	UV-Vis
32	p-NP	0.001	0		hv	5.5	0.04	LCMS
33	p-NP	0.001	3.7		hv	5.5	0.64	LCMS
34	p-NP	0.001	3.7		hv	2	1.26	LCMS
35	p-NP	0.001	0		hv	2	0.05	LCMS
36	Erythritol	1	0		hv	5	0.00	GCMS
37	Erythritol	1	3.7		dark	5	0.00	GCMS
38	Erythritol	1	3.7		hv	5	0.35	GCMS
39	Erythritol	1	0	200mM H_2O_2*	hv	5	0.45	GCMS
40	Erythritol	1	3.7	200mM H_2O_2	hv	5	0.66	GCMS
41	Pinonic Acid	1	0		hv	--	0.01	LCMS
42	Pinonic Acid	1	3.7		hv	--	0.30	LCMS
44	Pinonic Acid	1	3.7	MB AS, low light	hv	--	0.08	LCMS
45	Pinonic Acid	1	3.7	RG AS, low light	hv	--	0.10	LCMS
46	1,2-DHI	50	0		hv	--	1E-4	NMR
47	1,2-DHI	50§	3.7		hv	--	0.01	NMR
48	1,2-DHI	1	3.7		hv	--	0.53	Model
49	1,3,5-THB	1	0		hv	5	0.05	LCMS
50	1,3,5-THB	1	3.7		hv	5	0.96	LCMS
51	1,4 Dinitrate	1	0		hv	5.5	0.02	LCMS

52	1,4 Dinitrate	1	3.7	hv	5.5	0.14	LCMS
53	1,4 Dinitrate	1	0	hv	1.5	0.03	LCMS
54	1,4 Dinitrate	1	3.7	hv	2	0.13	LCMS

* Equivalent to 7 x 10^{-14} M of steady state [OH]

§ corrected to rate at 1mM concentration of organic using relationships in **Fig. 4.**

Table S2. Trace metals analysis of the Reagent Grade and Molecular Biology Grade ammonium sulfate (AS) reagents used in this work. Both grades are advertised by the manufacturer to have < 5 ppm of trace metals and < 5 ppm of Fe. Absolute concentrations of metals are measured in 0.15 M solutions of AS in ultrapure water, reported in ppb. Relative concentrations represent the mass fraction of metals in the material, obtained by dividing the absolute concentrations of metals by the concentration of the AS solution. The highest metallic contribution came from aluminum (Al) and iron (Fe) in the Reagent Grade AS at ~ 0.2-0.3 ppm relative to AS, which is much lower than the upper limit stated by the manufacturer.

**ABSOLUTE in 0.15 M solution**	**[Al] (ppb)**	**[Cr] (ppb)**	**[Mn] (ppb)**	**[Fe] (ppb)**	**[Co] (ppb)**	**[Ni] (ppb)**	**[Cu] (ppb)**	**[Zn] (ppb)**	**[As] (ppb)**	**[Ag] (ppb)**	**[Cd] (ppb)**	**[Pb] (ppb)**
AS Reagent Grade 20 g/L	6.027	1.040	0.265	3.815	0.011	0.961	0.314	**<0.67**	**<0.008**	0.010	0.001	0.159
AS Molec Bio Grade 20 g/L	**<0.93**	0.745	0.321	**<0.32**	**<0.005**	0.703	**<0.037**	**<0.67**	**<0.008**	**<0.001**	**<0.003**	0.015
**RELATIVE to AS mass**	**[Al] (ppm)**	**[Cr] (ppm)**	**[Mn] (ppm)**	**[Fe] (ppm)**	**[Co] (ppm)**	**[Ni] (ppm)**	**[Cu] (ppm)**	**[Zn] (ppm)**	**[As] (ppm)**	**[Ag] (ppm)**	**[Cd] (ppm)**	**[Pb] (ppm)**
AS Reagent Grade	0.3014	0.0520	0.0133	0.1907	0.0005	0.0481	0.0157	**<0.034**	**<0.0004**	0.0005	0.0001	0.0080
AS Molec Bio Grade	**<0.0465**	0.0373	0.0160	**<0.0160**	**<0.0003**	0.0352	**<0.0019**	**<0.034**	**<0.0004**	**<0.0001**	**<0.0002**	0.0007

Table S3. Photochemical reactions used in the kinetic model in addition to reaction data from JPL evaluation number 18.

**ORGANIC REACTIONS**	**Rate Coefficient (M-1 s-1)**	**Rate Coefficient (cm3 molec-1 s-1)**	**Reference**
erythritol + OH → Products	1.90E+09	3.16E-12	Hermann 2010
erythritol + SO_4 → Products	4.20E+07	6.98E-14	Hermann 2010
p-nitrobenzaldehyde + OH → Products	3.00E+09	4.98E-12	Hermann 2010
p-nitrobenzaldehyde + SO_4 → Products	4.50E+08	7.48E-13	Hermann 2010
tbutanol + OH → Products	7.00E+08	1.16E-12	Hermann 2010
tbutanol + SO_4 → Products	9.00E+05	1.50E-15	Hermann 2010
methanol + OH → Products	8.00E+08	1.33E-12	Hermann 2010
methanol + SO_4 → Products	1.00E+07	1.66E-14	Hermann 2010

**EQUILIBRIA**	**Rate Coefficient (M-1 s-1)**	**Rate Coefficient (cm3 molec-1 s-1)**	**Reference**
$H_2O \rightarrow H^+ + OH^-$	2.34E-05	3.89E-26	CAPRAM 1999
H+ + OH- → H_2O	1.30E+11	2.16E-10	CAPRAM 1999
$HO_2 \rightarrow O_2^- + H^+$	8.00E+05	1.33E-15	CAPRAM 1999
$O_2^- + H^+ \rightarrow HO_2$	5.00E+10	8.31E-11	CAPRAM 1999
$HSO_4^- \rightarrow SO_4^{2-} + H^+$	1.02E+09	1.69E-12	CAPRAM 1999
$SO_4^{2-} + H^+ \rightarrow HSO_4^-$	1.00E+11	1.66E-10	CAPRAM 1999

**INORGANIC REACTIONS**	**Rate Coefficient (M-1 s-1)**	**Rate Coefficient (cm3 molec-1 s-1)**	**Reference**
$OH + H_2O_2 \rightarrow H_2O + HO_2$	3.00E+07	4.98E-14	CAPRAM 1999
$OH + HO_2 \rightarrow H_2O + O_2$	1.00E+10	1.66E-11	CAPRAM 1999
$HO_2 + HO_2 \rightarrow O_2 + H_2O_2$	8.30E+05	1.38E-15	CAPRAM 1999
$HO_2 + O_2^-$ —(H^+)→ $H_2O_2 + O_2$	9.70E+07	1.61E-13	CAPRAM 1999
$OH + O_2^- \rightarrow OH^- + O_2$	1.10E+10	1.83E-11	CAPRAM 1999
$OH + HSO_4^- \rightarrow SO_4 + H_2O$	3.50E+05	5.81E-16	CAPRAM 1999
$SO_4 + SO_4 \rightarrow S_2O_8^-$	1.60E+08	2.66E-13	CAPRAM 1999
**$SO_4 + H_2O_2 \rightarrow SO_4^{2-} + H^+ + HO_2$**	1.20E+07	1.99E-14	Wine JGR 1989
$SO_4 + HO_2 \rightarrow SO_4^{2-} + H^+ + O_2$	3.50E+09	5.81E-12	CAPRAM 1999
$SO_4 + O_2^- \rightarrow SO_4^{2-} + O_2$	3.50E+09	5.81E-12	CAPRAM 1999
SO_4 + OH- → SO4^{2-} + OH	1.40E+07	2.33E-14	CAPRAM 1999
$SO_4 + H_2O \rightarrow$ SO4^{2-} + H^+ + OH (in s^{-1})	11.00	1.83E-20	CAPRAM 1999

**PHOTOLYSES**	**Aqueous Cross Section**	**Radical Quantum Yield**	**Note**
$H_2O_2 \rightarrow 2\,OH$	Chu & Anastasio 2005	(0.975) Chu & Anastasio 2005	Assumed in dilute solution, 298 K

Table S4. Calculations for scavenger kinetic experiments shown in **Figure 2A** of the main text.

**Rate coefficient**	**Value**	**Units**	**Reference**
t-butanol + OH	7.00E+08	M^{-1} s^{-1}	(2)
t-butanol + SO_4	9.00E+05	M^{-1} s^{-1}	(17)
methanol + OH	8.00E+08	M^{-1} s^{-1}	(2)
methanol + SO_4	1.00E+07	M^{-1} s^{-1}	(18)
p-Nitrophenol + OH	6.20E+09	M^{-1} s^{-1}	(2)
p-Nitrophenol + SO_4	6.60E+08	M^{-1} s^{-1}	(19)
**Scavenger**	**Methanol**		
Scavenger conc.	0.3	M	
Reactant conc.	0.001	M	
**% of SO_4 scavenged**	**82.0**		
**% of OH scavenged**	**97.5**		
**Scavenger**	**t-Butanol**		
Scavenger conc.	0.1	M	
Reactant conc.	0.001	M	
**% of SO_4 scavenged**	**12.0**		
**% of OH scavenged**	**91.9**		

Table S5. Observed and assigned peaks in the 1,2-DHI + AS + hv bulk aqueous experiment. Peak assignments were constrained to 0 ≤ S ≤ 1 and N = 0.

Exact m/z	S/N	C	H	H(neutral)	O	N	S
117.0195	4.88	4	5	6	4	0	0
129.0192	31.54	5	5	6	4	0	0
131.0351	15.71	5	7	8	4	0	0
132.9964	12.28	4	5	6	3	0	1
136.9911	13.86	3	5	6	4	0	1
138.9706	7.18	2	3	4	5	0	1
140.9862	7.51	2	5	6	5	0	1
145.0504	85.83	6	9	10	4	0	0
151.0066	6.16	4	7	8	4	0	1
152.9864	5.71	3	5	6	5	0	1
156.9814	48.16	2	5	6	6	0	1
160.9913	13.1	5	5	6	4	0	1
163.0069	13.95	5	7	8	4	0	1
165.0222	45.29	5	9	10	4	0	1
167.0016	35.89	4	7	8	5	0	1
175.0071	17.82	6	7	8	4	0	1
182.9965	32.43	4	7	8	6	0	1
183.0330	5.41	5	11	12	5	0	1
193.0177	184.32	6	9	10	5	0	1
195.0326	517.23	6	11	12	5	0	1
197.0124	8.06	5	9	10	6	0	1
197.0281	10.16	9	9	10	3	0	1
199.0277	4.93	5	11	12	6	0	1
206.9963	10.24	6	7	8	6	0	1
207.0334	6.26	7	11	12	5	0	1
209.0118	30.95	6	9	10	6	0	1
210.9909	4.58	5	7	8	7	0	1
211.0279	15.79	6	11	12	6	0	1
213.0064	24.75	5	9	10	7	0	1
215.0224	28.04	5	11	12	7	0	1
235.0644	15.4	9	15	16	5	0	1
237.0793	3.23	9	17	18	5	0	1
247.0647	14.91	10	15	16	5	0	1
249.0801	21.5	10	17	18	5	0	1
251.0585	17.34	9	15	16	6	0	1
253.0744	9.37	9	17	18	6	0	1
263.0202	5.06	12	7	8	7	0	0
263.0589	5.68	10	15	16	6	0	1
265.0745	235.86	10	17	18	6	0	1
265.1479	3.11	12	25	26	4	0	1
267.0539	54.17	9	15	16	7	0	1
267.0704	5.32	13	15	16	4	0	1
267.0902	120.31	10	19	20	6	0	1
279.0535	7.14	10	15	16	7	0	1
279.0905	4.93	11	19	20	6	0	1
281.0334	3.38	9	13	14	8	0	1
281.0690	34.61	10	17	18	7	0	1
283.0487	3.98	9	15	16	8	0	1
283.0847	12.81	10	19	20	7	0	1
297.0645	43.03	10	17	18	8	0	1
299.0811	33.45	10	19	20	8	0	1
333.1010	3.2	14	21	22	7	0	1
337.1323	5.28	14	25	26	7	0	1

349.1321	37.52	15	25	26	7	0	1
351.1473	25.42	15	27	28	7	0	1
365.1277	8.06	15	25	26	8	0	1
367.1424	23.39	15	27	28	8	0	1
381.1241	4.92	11	25	26	14	0	0
383.1395	4.16	15	27	28	9	0	1

Table S6. All observed HRMS peaks from the 1,2-DHI + AS dark chamber experiment after subtracting the AS only control**.** Peak assignments were constrained to $0 \leq S \leq 1$ and $N = 0$. Approximately 85% of peaks remain unassigned; presumably they are inorganic, multiply charged, or transient species. The observed peaks from C_5 compounds may indicate a possibility of reduced-sulfur-mediated dark reactions that occurred to a minor extent at the particle interface (*21*).

m/z	S/N	C	H	H(neutral)	O	N	S
124.9915	13.14	2	5	6	4	0	1
131.0351	6.07	5	7	8	4	0	0
212.9801	6.06	15	1	2	0	0	1
217.0111	3.15	15	5	6	0	0	1
265.1489	32.21	12	25	26	4	0	1
293.1805	9.55	14	29	30	4	0	1
297.1547	7.27	12	25	26	8	0	0
337.2060	4.34	16	33	34	5	0	1
339.2014	15.75	15	31	32	8	0	0
353.2014	10.72	16	33	34	6	0	1
381.2321	3.16	18	37	38	6	0	1
421.2276	14.74	20	37	38	7	0	1
431.0732	4.63	28	15	16	3	0	1

Table S7. All observed HRMS peaks from the 1,2-DHI + AS + hv chamber experiment after subtracting the dark experimental control (1,2-DHI + AS). Peak assignments were constrained to 0 ≤ S ≤ 1 and N = 0. Many peaks with negative mass defects that may be indicative of sulfur in the composition remain unassigned. The most abundant peak m/z 215.02 (shown in bold) is the trihydroxy organosulfate of isoprene.

m/z	S/N	C	H	H(neutral)	O	N	S
113.0243	4.2	5	5	6	3	0	0
118.9923	27.22	---	---	---	---	---	---
119.0350	3.03	4	7	8	4	0	0
124.0166	3.42	---	---	---	---	---	---
124.9551	5.89	1	1	2	5	0	1
125.8983	5.27	---	---	---	---	---	---
127.1124	4.41	8	15	16	1	0	0
127.8969	3.7	---	---	---	---	---	---
129.1284	5.89	8	17	18	1	0	0
130.9926	9.83	---	---	---	---	---	---
130.9982	3.43	4	3	4	5	0	0
133.0504	4.24	5	9	10	4	0	0
135.0662	4.29	5	11	12	4	0	0
138.9701	4.69	2	3	4	6	0	1
145.0142	4.91	5	5	6	5	0	0
147.0292	4.85	5	7	8	5	0	0
152.9859	11.06	3	5	6	5	0	1
155.0173	3.62	7	7	8	2	0	1
159.0298	3.06	6	7	8	5	0	0
165.0193	3.18	8	5	6	4	0	0
167.0020	7.94	4	7	8	5	0	1
168.9890	16.19	---	---	---	---	---	---
172.8295	3.75	---	---	---	---	---	---
173.1184	13.28	9	17	18	3	0	0
176.8433	9.63	---	---	---	---	---	---
178.8427	8.85	---	---	---	---	---	---
180.8394	5.7	---	---	---	---	---	---
180.9893	6.26	---	---	---	---	---	---
182.9960	18.14	4	7	8	6	0	1
184.9840	101.54	---	---	---	---	---	---
186.9050	20.52	---	---	---	---	---	---
188.9033	9.39	---	---	---	---	---	---
194.9880	8.65	---	---	---	---	---	---
197.0120	8.52	5	9	10	6	0	1
197.8517	6.11	---	---	---	---	---	---
201.1126	12.38	10	17	18	4	0	0
201.6904	4.25	---	---	---	---	---	---
202.0459	3.95	---	---	---	---	---	---
202.1479	20.26	---	---	---	---	---	---
202.2468	4.48	---	---	---	---	---	---
213.0071	6.37	5	9	10	7	0	1
214.0565	4.39	---	---	---	---	---	---
**215.0231**	**78.03**	**5**	**11**	**12**	**7**	**0**	**1**
215.0922	4.66	10	15	16	5	0	0
217.0014	3.12	4	9	10	8	0	1
218.9863	29.05	---	---	---	---	---	---
230.9859	3.27	---	---	---	---	---	---
231.9427	63.59	---	---	---	---	---	---
232.9887	10.17	14	1	2	4	0	0
243.0461	14.1	17	7	8	2	0	0

244.9848	7.04	---	---	---	---	---	---
248.8925	3.86	---	---	---	---	---	---
257.0325	6.93	7	13	14	8	0	1
266.9198	3.73	---	---	---	---	---	---
266.9676	3.19	---	---	---	---	---	---
267.0104	3.18	---	---	---	---	---	---
268.9832	4.47	---	---	---	---	---	---
271.0488	8.25	8	15	16	8	0	1
278.9701	4.69	---	---	---	---	---	---
283.8252	3.1	---	---	---	---	---	---
299.0803	8.41	10	19	20	8	0	1
300.0010	6.71	---	---	---	---	---	---
311.1676	51.24	17	27	28	3	0	1
312.9716	4.49	---	---	---	---	---	---
318.9799	5.19	---	---	---	---	---	---
325.1828	41.06	18	29	30	3	0	1
326.1866	6.96	---	---	---	---	---	---
327.1035	4	22	15	16	3	0	0
328.0894	4.08	---	---	---	---	---	---
332.1189	16.38	---	---	---	---	---	---
340.2021	4.02	---	---	---	---	---	---
344.0834	5.58	---	---	---	---	---	---
373.0733	3.63	22	13	14	6	0	0
384.9779	3.6	24	1	2	6	0	0
391.0837	3.51	22	15	16	7	0	0
398.1662	3.23	---	---	---	---	---	---
401.8970	43.67	---	---	---	---	---	---
412.1451	3.58	---	---	---	---	---	---
414.1601	4.66	---	---	---	---	---	---
421.2243	3.02	23	33	34	7	0	0
431.0699	3.44	10	23	24	16	0	1
469.9549	3.04	---	---	---	---	---	---

Table S8. Extrapolations to atmospheric oxidation rates (R, in μM/h**) based on atmospheric vs. laboratory experiments.** Empirical ratios between lab and atmosphere were presented in **Figure 1**, using p-NP as a model compound. The first-order decay rates (k_{obs}) of p-NP measured under summer noon-time and clear sky conditions **(Fig. S4C**) were used in order to scale other rates to estimated "daytime" values from their lab determinations. Direct photolysis controls have been subtracted from all rates. First-order k_{obs} are based on data using 3.7 M AS and 1 mM of organics; average k_{obs} assume an average light intensity between day and night. The total rate of oxidation is not expected to be dependent on organic concentration (**Fig. 4**).

Compound Name	Lab k_{obs} (h^{-1})	Atmospheric Avg. k_{obs} (h^{-1})	Atm. daytime k_{obs} (h^{-1})	Atm. Avg. lifetime (day)	Daytime Rate of oxidation (uM h^{-1})
Dinitrate	0.12	0.02	0.05	1.7	49
p-NP	0.17	0.03	0.07	1.2	70
pinonic	0.30	0.06	0.12	0.7	119
erythritol	0.35	0.07	0.14	0.6	143
1,2-DHI	0.51	0.10	0.21	0.4	209
THB	0.91	0.19	0.37	0.2	373
DNPH	0.41	0.08	0.17	0.5	168
FADNPH	0.12	0.02	0.05	1.7	49

SI References.

1. A. Tilgner, H. Herrmann, "Tropospheric Aqueous-Phase OH Oxidation Chemistry: Current Understanding, Uptake of Highly Oxidized Organics and Its Effects" in Multiphase Environmental Chemistry in the Atmosphere. (ACS Publications, 2018), pp. 49-85.
2. H. Herrmann, D. Hoffmann, T. Schaefer, P. Bräuer, A. Tilgner, Tropospheric aqueous-phase free-radical chemistry: Radical sources, spectra, reaction kinetics and prediction tools. *ChemPhysChem* **11**, 3796-3822 (2010).
3. C. Anastasio, J. T. Newberg, Sources and sinks of hydroxyl radical in sea-salt particles. *J. Geophys. Res.* **112** (2007).
4. T. Arakaki *et al.*, A General Scavenging Rate Constant for Reaction of Hydroxyl Radical with Organic Carbon in Atmospheric Waters. *Environ. Sci. Technol.* **47**, 8196-8203 (2013).
5. R. Kaur *et al.*, Photooxidants from brown carbon and other chromophores in illuminated particle extracts. *Atmos. Chem. Phys.* **19**, 6579-6594 (2019).
6. Y. Tang, R. P. Thorn, R. L. Mauldin, P. H. Wine, Kinetics and spectroscopy of the SO4− radical in aqueous solution. *Journal of Photochemistry and Photobiology A: Chemistry* **44**, 243-258 (1988).
7. S. P. Sander *et al.*, *Chemical kinetics and photochemical data for use in atmospheric studies evaluation number 15* (JPL Publication 06-2, Jet Propulsion Laboratory, Pasadena, 2006 http://jpldataeval.jpl.nasa.gov).
8. J. D. Crounse, K. A. McKinney, A. J. Kwan, P. O. Wennberg, Measurement of gas-phase hydroperoxides by chemical ionization mass spectrometry. *Anal. Chem.* **78**, 6726-6732 (2006).
9. L. Chu, C. Anastasio, Quantum Yields of Hydroxyl Radical and Nitrogen Dioxide from the Photolysis of Nitrate on Ice. *J. Phys. Chem. A* **107**, 9594-9602 (2003).
10. L. Chu, C. Anastasio, Formation of Hydroxyl Radical from the Photolysis of Frozen Hydrogen Peroxide. *J. Phys. Chem. A* **109**, 6264-6271 (2005).
11. P. Podolec *et al.*, Direct silylation of Trypanosoma brucei metabolites in aqueous samples and their GC–MS/MS analysis. *Journal of Chromatography B* **967**, 134-138 (2014).
12. Y. Kanaya, Y. Kajii, H. Akimoto, Solar actinic flux and photolysis frequency determinations by radiometers and a radiative transfer model at Rishiri Island: comparisons, cloud effects, and detection of an aerosol plume from Russian forest fires. *Atmos. Environ.* **37**, 2463-2475 (2003).
13. H. Ren, J. A. Sedlak, M. J. Elrod, General Mechanism for Sulfate Radical Addition to Olefinic Volatile Organic Compounds in Secondary Organic Aerosol. *Environ. Sci. Technol.* **55**, 1456-1465 (2021).
14. L. Wojnárovits, E. Takács, Rate constants of sulfate radical anion reactions with organic molecules: A review. *Chemosphere* **220**, 1014-1032 (2019).
15. G. S. Tyndall *et al.*, Atmospheric chemistry of small organic peroxy radicals. *J. Geophys. Res.* **106**, 12157-12182 (2001).
16. J. J. Orlando, G. S. Tyndall, T. J. Wallington, The Atmospheric Chemistry of Alkoxy Radicals. *Chem. Rev.* **103**, 4657-4689 (2003).
17. C. George, H. E. Rassy, J.-M. Chovelon, Reactivity of selected volatile organic compounds (VOCs) toward the sulfate radical (SO4−). *Int. J. Chem. Kinet.* **33**, 539-547 (2001).
18. S. Padmaja, Z. Alfassi, P. Neta, R. Huie, Rate constants for reactions of SO4− radicals in acetonitrile. *Int. J. Chem. Kinet.* **25**, 193-198 (1993).
19. K. J. Rudziński, R. Szmigielski, Aqueous Reactions of Sulfate Radical-Anions with Nitrophenols in Atmospheric Context. *Atmosphere* **10**, 795 (2019).
